# Impact of zinc supplementation on phenotypic antimicrobial resistance of fecal commensal bacteria from pre-weaned dairy calves

**DOI:** 10.1038/s41598-024-54738-x

**Published:** 2024-02-23

**Authors:** Katie Y. Lee, Edward R. Atwill, Xunde Li, Hillary R. Feldmann, Deniece R. Williams, Bart C. Weimer, Sharif S. Aly

**Affiliations:** 1 Department of Population Health and Reproduction, School of Veterinary Medicine, University of California, Davis, Davis, CA USA; 2 Veterinary Medicine Teaching and Research Center, University of California Davis, Tulare, CA USA

**Keywords:** Antimicrobial resistance, Infectious-disease epidemiology

## Abstract

The objective of this study was to evaluate the impact of dietary zinc supplementation in pre-weaned dairy calves on the phenotypic antimicrobial resistance (AMR) of fecal commensal bacteria. A repository of fecal specimens from a random sample of calves block-randomized into placebo (n = 39) and zinc sulfate (n = 28) groups collected over a zinc supplementation clinical trial at the onset of calf diarrhea, calf diarrheal cure, and the last day of 14 cumulative days of zinc or placebo treatment were analyzed. Antimicrobial susceptibility testing was conducted for *Enterococcus* spp. (n = 167) and *E. coli* (n = 44), with one representative isolate of each commensal bacteria tested per sample. Parametric survival interval regression models were constructed to evaluate the association between zinc treatment and phenotypic AMR, with exponentiated accelerated failure time (AFT) coefficients adapted for MIC instead of time representing the degree of change in AMR (MIC Ratio, MR). Findings from our study indicated that zinc supplementation did not significantly alter the MIC in *Enterococcus* spp. for 13 drugs: gentamicin, vancomycin, ciprofloxacin, erythromycin, penicillin, nitrofurantoin, linezolid, quinupristin/dalfopristin, tylosin tartrate, streptomycin, daptomycin, chloramphenicol, and tigecycline (MR = 0.96–2.94, *p* > 0.05). In *E. coli*, zinc supplementation was not associated with resistance to azithromycin (MR = 0.80, *p* > 0.05) and ceftriaxone (MR = 0.95, *p* > 0.05). However, a significant reduction in *E. coli* MIC values was observed for ciprofloxacin (MR = 0.17, 95% CI 0.03–0.97) and nalidixic acid (MR = 0.28, 95% CI 0.15–0.53) for zinc-treated compared to placebo-treated calves. Alongside predictions of MIC values generated from these 17 AFT models, findings from this study corroborate the influence of age and antimicrobial exposure on phenotypic AMR.

## Introduction

Antimicrobial resistance (AMR) is one of the most significant public health threats faced in this century^1^. The global challenge to address AMR has prompted studies to better ascertain its distribution across diverse host populations and environments, to infer the pathways of its spread, and to identify the risk factors associated with its emergence and persistence. Comparably, understanding of specific factors that modulate AMR and the extent to which they promote or decrease resistance, particularly in food-producing animals, remains limited.

The occurrence of AMR in food-producing animals not only limits therapeutic options that compromises animal health, but also raises concerns of the potential for its dissemination through animal populations, environmental matrices, and the food chain^2^. In dairy cattle, it has been well documented that a higher frequency of AMR is typically observed in calves compared to older cattle^3–6^. This observed difference in resistance between younger and older cattle may partially be attributed to factors such as increased susceptibility to disease during early life, dietary changes, and initial exposure to the environment and antimicrobial treatments^7,8^. Improving our understanding of AMR dynamics during early calf life is thus of considerable importance as this period presents an opportunity to reduce AMR acquisition in bovine hosts and their environments.

Diarrhea is the leading cause of morbidity and mortality in pre-weaned dairy calves, with causative agents including viral (e.g. bovine rotavirus), parasitic (e.g. *Cryptosporidium parvum*), and bacterial pathogens (e.g. *Salmonella enterica*)^9^. In diarrheic calves, the frequency of co-infection with more than one pathogen and increased susceptibility to bacteremia from compromised small intestinal function makes antimicrobial therapy a critical and frequent component of treatment protocols^10,11^; the USDA reported in 2014 that 21.1% of pre-weaned heifers were affected with diarrhea or other digestive disorders, of which 75.9% were treated with antimicrobials^12^. To ensure animal health and mitigate AMR, alternatives such as zinc supplementation has been explored^13,14^. Zinc is a trace mineral with a crucial role in many aspects of cellular metabolism and a key constituent of over 300 structural or catalytic enzymatic functions^15^. It also has an essential role in cellular signal transduction, cell proliferation and differentiation, and regulation of innate and adaptive immunity^15,16^. Deficiency compromises mechanisms involved in pathogen neutralization and alters cytokine production to impact inflammatory responses, resulting in increased risk for disease and infection in hosts^15^. The immunity and gut integrity benefits of zinc makes its application in dairy calves of particular interest to simultaneously reduce incidence of diarrheal diseases, improve calf health and growth, and decrease antimicrobial use^13,17–19^.

In a previously conducted double-blind, block-randomized, placebo-controlled clinical trial, zinc supplementation was shown to be effective in delaying diarrheal onset and expediting diarrhea recovery^14^. However, the utility of zinc supplementation may have unintended consequences as heavy metal exposure has been suggested to enhance the spread of AMR due to the genetic and physiological linkages between metal resistance and AMR. Mechanistically, co-selection for metal resistance and AMR could putatively arise through cross-resistance (one gene/mechanism confers both metal resistance and AMR), co-regulation (the expression of transcriptionally linked metal resistance and AMR systems are modulated by a common gene/regulator), and/or co-resistance (metal resistance and AMR genes occur on the same genetic element)^20^. Studies evaluating the relationship between dietary zinc and AMR have primarily focused on swine, with several studies providing supporting evidence for the selection of multidrug resistance and increase in occurrence and abundance of AMR genetic determinants from zinc supplementation^21–24^. In bovine hosts, studies on dietary zinc and AMR have focused on beef cattle with mixed findings of no AMR selection^25,26^ or increased AMR to certain antimicrobials in enteric bacteria^27^. Currently, little is known on the effect of dietary zinc supplementation on AMR in calves. Additionally, the influence on AMR from other contributing factors in conjunction with dietary zinc remains unclear.

To address these knowledge gaps, the aim of this study was to evaluate the impact of dietary zinc supplementation in pre-weaned dairy calves on the phenotypic AMR of fecal commensal bacteria. The objectives were to determine the association between zinc supplementation and AMR in *E. coli* and *Enterococcus* spp. and quantify changes in resistance with respect to dietary zinc exposure and calf-level factors.

## Materials and methods

The original trial procedures that generated the repository of fecal samples for the current study were approved by the University of California Davis Institutional Animal Care and Use Committee (protocol number 18067 Approved: March 6, 2014) and performed in accordance with relevant guidelines and regulations. The trial that generated the sample repository was a double-blind, block randomized, placebo-controlled clinical trial evaluating different dietary zinc supplementation treatments and their effect on diarrhea prevention and health in pre-weaned calves. The trial was conducted on a single San Joaquin Valley dairy (Kings County, CA, USA). The detailed study and sampling design were previously described^14^. Briefly, healthy Holstein heifer or bull calves from the one dairy were enrolled at birth (24–48 h of age) and block-randomized by time of birth to zinc or placebo treatment groups. Calves were examined by a veterinarian or trained personnel and excluded if exhibiting obvious morbidities or congenital defects^14^. A random sample of enrolled calves (approximately 8–10% of the study population given budgetary constraints for sample testing) were selected for fecal sampling. Calves analyzed for this study included all sampled calves (n = 67) in the zinc sulfate (n = 28) and placebo (n = 39) treatment groups that had onset of diarrhea at some point over the duration of the clinical trial.

### Pre-weaned calf management and treatment protocols

All calves were under the same management practices including housing and diet^14^. Throughout the study, calves were housed in individual metal hutches. The respective treatments were administered in the morning milk bottles for 14 days starting from enrollment; calves in the zinc sulfate treatment group received 0.22 g zinc sulfate monohydrate (80 mg of elemental zinc) (Sigma-Aldrich Company, St. Louis, MO, United States) with 0.44 g milk replacer powder, while those in the placebo group received only milk replacer powder (0.44 g). Antimicrobials included in dietary milk for calves 0–25 days of age included chlortetracycline hydrochloride (Pennchlor 64, Pharmgate Animal Health, Omaha, NE, United States) and neomycin sulfate (NeoMed 325, Bimeda, Inc., Le Sueur, MN, United States). Calves 25 days of age to weaning received dietary milk that included oxytetracycline hydrochloride (NT-10G, Agri-Best™, Strauss Feeds LLC, Watertown, WI, United States). Calves from which samples were employed for the current study were all treated for symptoms of diarrhea at some point over the duration of the trial and received the same treatment consisting of an oral mixture of 118.5 mL bismuth subsalicylate (Bismusal Suspension, Durvet, Inc., Blue Springs, MO, United States) and 31.5 mL of spectinomycin (SpectoGard, Bimeda, Inc., Le Sueur, MN, United States)^14^.

### Fecal sample collection

Fecal samples were collected on the first day of calf diarrhea onset (D1), exit or cure from diarrhea (Dex), and on the last day of the 14-day treatment period (D14). Sampling was conducted only at two time points for calves where onset of diarrhea occurred on the same day as the last day of the treatment period (D1,D14). Sample collection was conducted as previously described; new gloves and sterile lubricant were used to collect fresh feces (~ 5 g) into 20 mL polypropylene jars (The Cary Company, QAddison, IL) by digital rectal simulation^14^. Fecal samples were stored at − 20 °C until analysis.

### Bacterial isolation

Fecal samples were processed for bacterial isolation by enriching a saturated fecal cotton swab in 10 mL of Tryptic Soy Broth (TSB, Becton Dickinson, Franklin Lakes, NJ, United States) for 24 h. Two 1 mL aliquots from each TSB enrichment were transferred to two tubes, one containing 9 mL of MacConkey and another containing 9 mL of Enterococcosel broth and were incubated for 24 h at 35 °C and 45 °C, respectively. A loopful (10 µL) of MacConkey and Enterococcosel broth was streaked to MacConkey and Enterococcosel agar for isolation of *E. coli* and *Enterococcus* spp., respectively. One putative colony of *E. coli* and *Enterococcus* spp. based on typical morphology (pink colony for *E. coli* and beige colony with strong black halo for *Enterococcus* spp.) was randomly selected from each selective agar plate, and streaked to purity by subculturing on respective selective agars then to blood agar plates (Tryptic Soy Agar with 5% sheep blood, Thermo Scientific, Waltham, MA, United States). All incubation steps for agar plates were performed at 35 ± 2 °C for 18–24 h.

### Bacterial confirmation

DNA was extracted from pure cultures of *E. coli* and *Enterococcus* spp. on blood agar plates using a boiling method. Briefly, colonies were suspended in 100 µL of molecular grade water in a sterile 1.5 mL microcentrifuge tube, incubated on a heating block at 100 °C for 20 min, and then centrifuged for 10 min at 5000 rpm^28^. Confirmation of isolates was then conducted as previously described using conventional PCR to screen for the presence of universal stress protein *uspA* for *E. coli*^29,30^, and 23S rRNA sequence using forward (ECST784F) and reverse (ENC854R) primers for *Enterococcus* spp^31,32^. Primer sequences used are provided in Supplementary Table 1. During the PCR step, a positive control (*E. coli* ATCC 25922 or *Enterococcus faecalis* ATCC 29212) and two negative controls comprised of a molecular grade water and PCR mastermix blank were used. Confirmed *E. coli* and *Enterococcus* spp. isolates were stored in cryovials containing TSB with 15% glycerol at − 80 °C for further testing.

### Antimicrobial susceptibility testing (AST)

Antimicrobial susceptibility testing (AST) was conducted on *E. coli* isolates from D14 fecal samples and 167 enterococci isolates from fecal samples collected at all sample collection time points using the NARMS Gram negative (YCMV3AGNF) and Gram positive (YCMV3AGPF) AST panels, respectively^5^. Briefly, 3–5 colonies of fresh, pure culture were inoculated into 5 mL of dH2O. Turbidity of the suspension was adjusted to 0.5 McFarland standard, or the equivalent of a 0.08–0.1 OD at 625 nm. From the suspension, 10 µL was then transferred to 11 mL of Sensititre Mueller–Hinton Cation adjusted broth to yield 1 × 10^5^ CFU/mL of bacterial suspension. Fifty microliters of the Mueller–Hinton inoculum were then transferred into each well of the AST plate and incubated at 35 °C for 24 h. *E. coli* ATCC 25922 and *Enterococcus faecalis* ATCC 29212 were used as quality controls. Minimum inhibitory concentration (MIC) values were recorded as the lowest concentration of each antimicrobial drug (AMD) which inhibited visible growth of bacteria. The antimicrobial drugs in each panel and their corresponding dilution ranges (µg/mL) are provided in Supplementary Table 2–3.

### Statistical analysis

- All statistical analyses were conducted using Stata 17.0. Data visualization was conducted in RStudio (ggplot2) and BioRender
- Calf-level factors

Descriptive statistics of enrolled calves for this study and the characteristics of *E. coli* and *Enterococcus* spp.—with each isolate representing the status of a calf from which the fecal isolate was collected from—were determined based on daily assessment records of individual calves. This included treatment group (whether an isolate originated from a fecal sample collected from a placebo or zinc sulfate treated calf), and calf-level factors of age, diarrhea status, and therapeutic spectinomycin exposure at the time of fecal collection. Different specifications of calf-level covariates were evaluated. For the age of the calf, this included age in days and age by week. Diarrhea status was specified as days on or from diarrhea (positive values indicating days of ongoing diarrhea and negative values indicating days post diarrhea recovery), categorical diarrhea status (isolate collected from a calf that was pre-diarrheic, diarrheic, or recovered from diarrhea). Exposure to spectinomycin was specified as the number of days from the most recent treatment, the number of doses received, whether treatment was received or not, and a categorical variable of days from the most recent treatment (*E. coli*: 0 days, 3–5 days, 6–8 days, and 9–10 days; *Enterococcus* spp.: 0 days, 1–3 days, 4–7 days, 8–23 days).

c. Accelerated failure time (AFT) models

To evaluate the association between isolates from zinc/placebo treatment groups and the degree of change in MIC values, an accelerated failure time (AFT) model was constructed for each antimicrobial drug. The AFT model is a parametric survival model that can be represented by:

$$ln\left( {{\text{T}}_{{\text{i}}} } \right) =\upbeta _{0} + {\text{X}}_{{\text{i}}}\upbeta _{{\text{i}}} + \varepsilon_{{\text{i}}}$$

For this study, the AFT model was adapted such that the dependent variable T_i_, typically representing time-to-event, is specified as left (≤ AMD_min_ μg/mL), right (> AMD_max_ μg/mL), or interval censored (between two twofold tested antimicrobial drug concentrations) MIC data, where AMD_min_ and AMD_max_ represent the lowest and highest antimicrobial drug concentrations tested, respectively. As negative MIC values are not biologically possible, all left-censored MIC values were specified with a lower bound of 0 instead of − ∞. The relationship between T_i_—the progression in expected concentration to inhibition for an antimicrobial for the *i*th isolate—and a random error term (ε_i_) are assumed to follow a specified distribution. Fixed effects are denoted by the intercept (β_0_) and X_i_β_i_ representing covariates (X_i_) and their corresponding regression coefficients (β_i_). To account for confounding from the biological influence of age and therapeutic antimicrobial treatment for diarrhea, calf age and exposure to spectinomycin were controlled for by forced inclusion as independent variables in all *Enterococcus* spp. models. Additionally, repeated measures from *Enterococcus* spp. collected at multiple time points were accounted for by calculating robust standard error estimates using a clustered sandwich variance estimator, which relaxed the assumption of independence among observations and allowed for intragroup correlation of isolates from the same calf^33,34^. As all *E. coli* isolates evaluated were recovered from fecal samples collected at the last day of the 14-day zinc or placebo treatments since calf enrollment (1–2 days of age), all *E. coli* isolates corresponded to calves of similar age (14–16 days), and only exposure to spectinomycin was controlled for in all *E. coli* models.

d. AFT model specification

A manual forward model building approach was used, in which a base model with treatment group as the only independent variable was first evaluated based on BIC estimates to specify a best-fitting (lowest BIC) distribution (Weibull, exponential, or generalized gamma) for model building. The final model with addition of confounders (spectinomycin exposure for *E. coli* models and spectinomycin exposure and calf age for *Enterococcus* spp. models) was identified by evaluating the various specifications for calf-level covariates and using the method of change in estimates to assess confounding. Other calf-level covariates (e.g. diarrhea status) were evaluated and retained in the model if improving model fit. The significance of biologically relevant interactions, specifically calf age—which was collinear with the days of zinc/placebo treatment exposure—and treatment group was tested for all *Enterococcus* spp. models to assess if a dose-dependent treatment effect was present. The specified parametric distribution for each model was also re-evaluated after tentative multivariable models were constructed, with Wald tests used to confirm that the appropriate parametric model was selected. Lastly, the final model for each antimicrobial drug was selected on the criteria that it generated estimates within the hypothetical maximum concentration limit for the MIC (1 × 10^6^ µg/mL)^35,36^. In favor of more parsimonious model selection, the Bayesian Information Criteria (BIC) was used to assess competing models, with lower values indicating better model fit. A 5% significance level was used for all models.

## Results

### Study population and characteristics of *E. coli* and *Enterococcus* spp.

**Figure 1:**
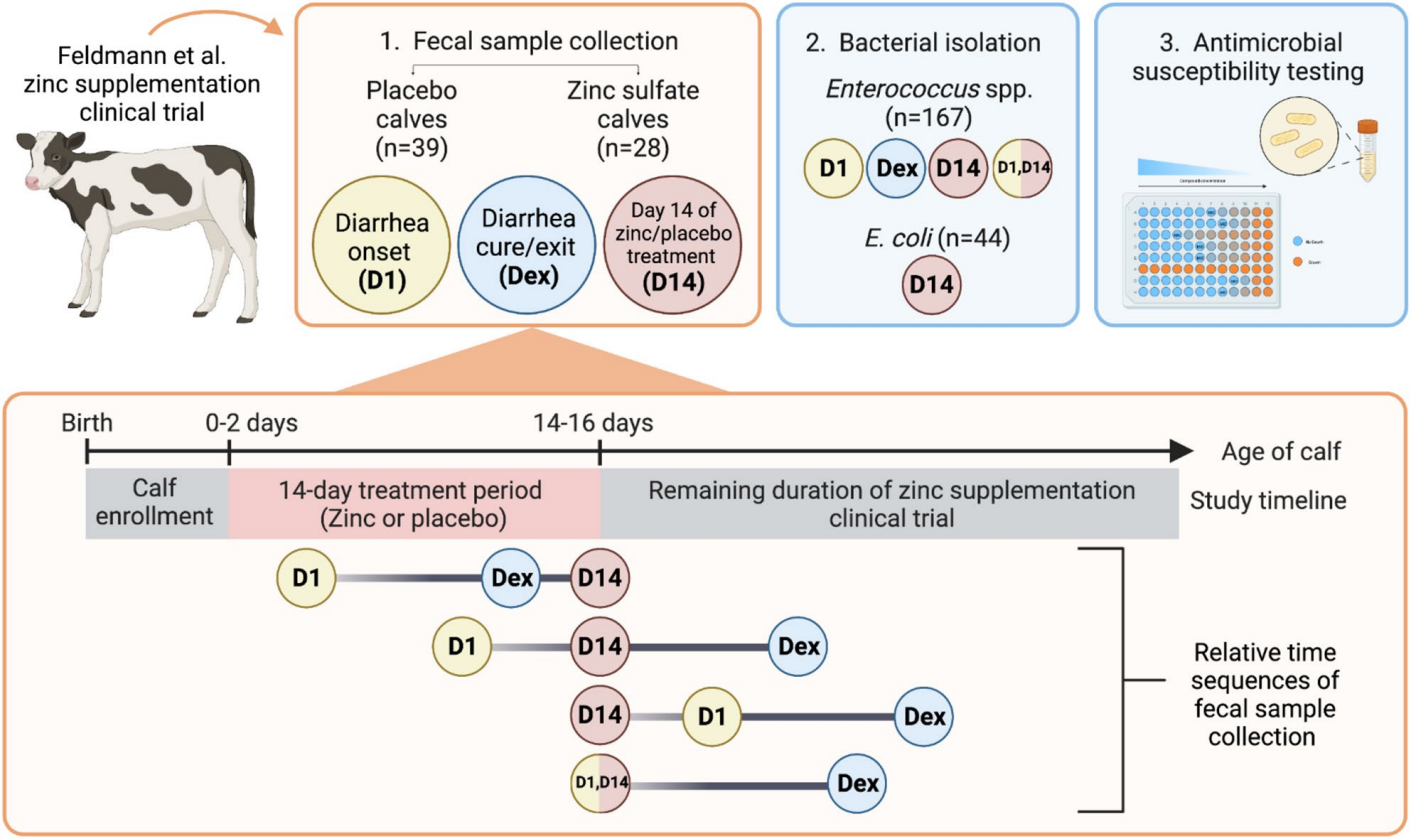
Schematic of fecal collection and analysis of samples collected from pre-weaned dairy calves enrolled in a zinc supplementation clinical trial.

Fecal samples from randomly selected pre-weaned dairy calves (28 calves from the zinc sulfate treatment group and 39 calves from the placebo treatment group) from a previously conducted zinc supplementation trial were assessed in this study^14^. Due to the observational nature of sample collection, fecal collection occurred across four possible time sequences as detailed in the study schematic (Fig. 1). Characteristics of pre-weaned dairy calves whose samples were identified for the current study are summarized in Table 1; no significant differences in sex, attitude score, fecal score, and age were observed between calves in the zinc sulfate and placebo treatment groups from which fecal samples were utilized in the present study.

**Table 1:** Characteristics of Holstein pre-weaned dairy calves from a zinc supplementation clinical trial randomly selected for fecal sample collection and analysis.

Characteristic	Zinc sulfate (n = 28)	Placebo (n = 39)	Total (n = 67)	*p*-value*
Sex				
Bull	14/28 (50%)	23/39 (59.0%)	37/67 (55.2%)	0.62
Heifer	14/28 (50%)	16/39 (41.0%)	30/67 (44.8%)	
Attitude score				
1 (Bright, alert, readily stood w/stimulation)	16/28 (57.1%)	31/39 (79.5%)	47/67 (70.1%)	0.06
2 (Quiet, alert, and stood only with moderate stimulation)	12/28 (42.9%)	8/39 (20.5%)	20/67 (29.9%)	
Fecal score				
1 (Solid)	21/28 (75%)	31/39 (79.5%)	52/67 (77.6%)	0.89
2 (Semi-formed/loose)	1/28 (3.6%)	2/39 (5.1%)	3/67 (4.5%)	
NS = None seen	6/28 (21.4%)	6/39 (15.4%)	12/67 (17.9%)	
Age at enrollment				
1 day	7/28 (25%)	9/28 (32.1%)	16/67 (23.9%)	1.00
2 days	21/28 (75%)	30/39 (76.9%)	51/67 (76.1%)	

*Fisher’s exact test.

**Figure 2:**
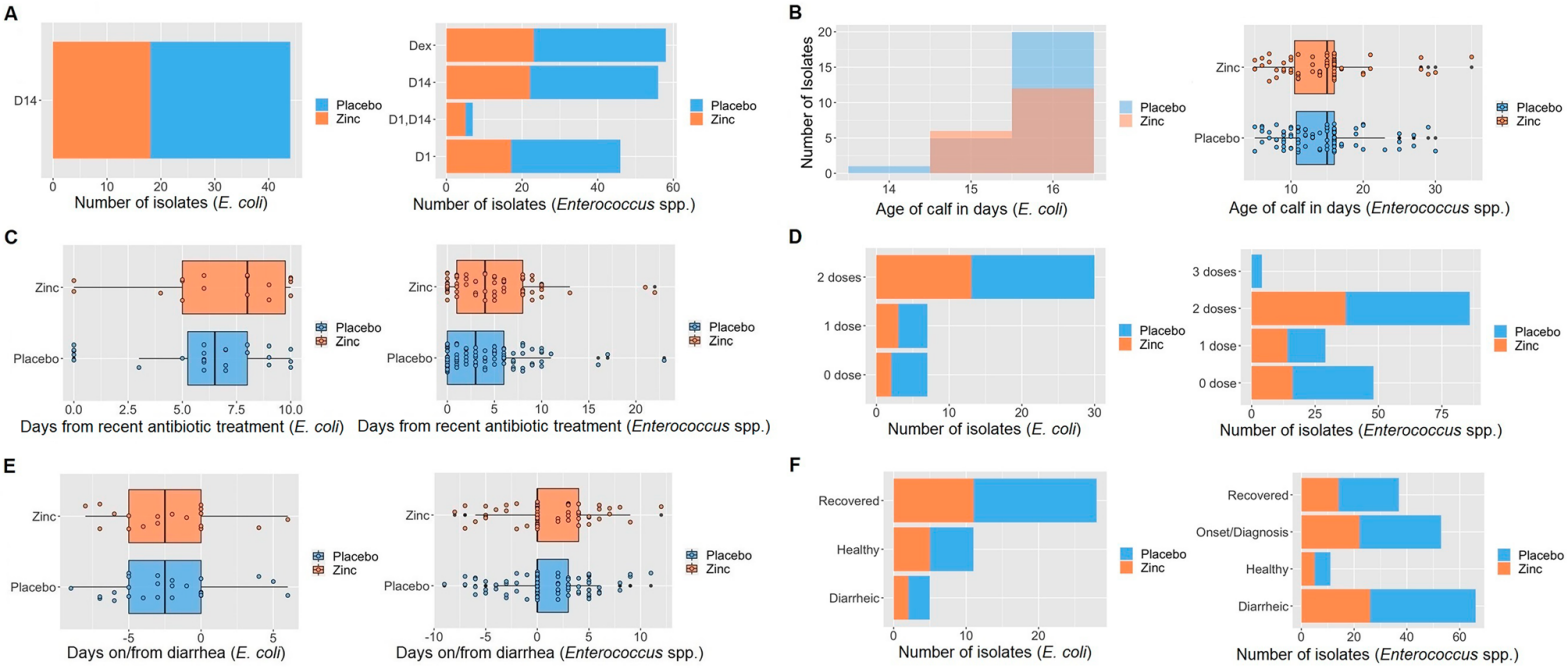
Characteristics of *E. coli* (n = 44) and *Enterococcus* spp. (n = 167) from pre-weaned dairy calves at the time of fecal sample collection by placebo and zinc treatment groups (**A**) Fecal sample type (**B**) Age of calf (**C**) Number of days from recent therapeutic spectinomycin treatment (**D**) Number of therapeutic spectinomycin treatments received (**E**) Days from diarrhea onset (positive numbers) and from diarrheal cure (negative numbers) and (**F**) Diarrhea disease status.

A total of 44 *E. coli* isolates from D14 fecal samples and 167 *Enterococcus* spp. from fecal samples collected at all of the aforementioned time points were included for antimicrobial susceptibility testing. Due to both budgetary constraints for laboratory testing and lower recovery of *E. coli* isolates from fecal samples collected at other time points, analysis of *E. coli* was restricted to isolates from D14 samples. As one representative isolate was tested per fecal sample, each commensal isolate represents the AMR status of a calf at the corresponding time point of fecal collection. Of the 44 *E. coli* isolates, 18 (40.91%) and 26 (59.09%) were from zinc and placebo treated calves, respectively. The average age of calves that *E. coli* isolates were collected from was 15.7 days (SD 0.51). Of the *E. coli* isolates, 15.91% corresponded to fecal samples from calves which received no spectinomycin treatment, 15.91% received 1 dose, and 68.18% received 2 doses. Further, 25%, 11.36%, and 63.64% of *E. coli* isolates were from healthy, diarrheic, and diarrhea-recovered calves, respectively. For the 167 enterococci isolates collected through repeated sampling of calves, 67 (40.12%) and 100 (59.88%) were from zinc and placebo treated calves, respectively. The average age of calves which enterococci isolates were collected from was 14.34 days (SD 5.47). Further, 28.74%, 17.37%, 51.50%, and 2.4% of enterococci isolates corresponded to calves having received 0, 1, 2, and 3 doses of spectinomycin treatment, respectively; while 6.59%, 31.74%, 39.52%, and 22.16% were from healthy, day of diarrhea diagnosis, diarrheic, and diarrhea-recovered calves, respectively. Characteristics of commensal isolates by treatment group are summarized in Fig. 2.

### Accelerated failure time (AFT) models

Due to the high frequency of right-censored antimicrobial susceptibility testing (AST) data, certain antimicrobial drugs were determined to be unsuitable for modelling given data sparseness and omitted from analysis. This included tetracycline, sulfisoxazole, trimethoprim-sulfamethoxazole, ampicillin, streptomycin, cefoxitin, chloramphenicol, amoxicillin-clavulanic acid, gentamicin, and ceftiofur for *E. coli*; and tetracycline, kanamycin, and lincomycin for enterococci isolates. The distribution of minimum inhibitory concentration (MIC) values and descriptive statistics (MIC_50_ and MIC_90_) for all tested drugs from AST for *E. coli* and *Enterococcus* spp. isolates are presented in Supplementary Figs. 1–6 and Supplementary Tables 4 and 5.

A total of 17 accelerated failure time (AFT) models, consisting of models for 4 antimicrobial drugs for *E. coli* (Supplementary Table 6–9) and 13 antimicrobial drugs for *Enterococcus* spp. (Supplementary Tables 10–22), were constructed. For *E. coli*, the ciprofloxacin model with spectinomycin exposure specified as the number of doses (BIC 189.5612) was selected as the final model over the competing model with a binary spectinomycin treated or not variable (BIC 188.6194) despite having a lower BIC in the latter given the significant coefficient for the number of doses variable (Supplementary Table 7). For *Enterococcus* spp., the generalized gamma model for streptomycin (BIC 260.1047, Supplementary Table 13) was selected over its Weibull model counterpart (BIC 249.5898) due to estimates from the latter model exceeding the hypothetical maximum concentration for MIC; for the same reason, the exponential model for gentamicin (BIC 524.5724, Supplementary Table 22) was selected as the final model over its Weibull model counterpart (BIC 268.565). Lastly, the *Enterococcus* spp. linezolid model with categorical number of days from last spectinomycin treatment (BIC 303.3108) was selected as the final model over the one with a binary spectinomycin (treated or not) variable (BIC 299.7655) given the significant coefficient for the categorical variable (Supplementary Table 16).

### Impact of zinc supplementation on AMR of fecal *E. coli* and *Enterococcus* spp.

**Figure 3:**
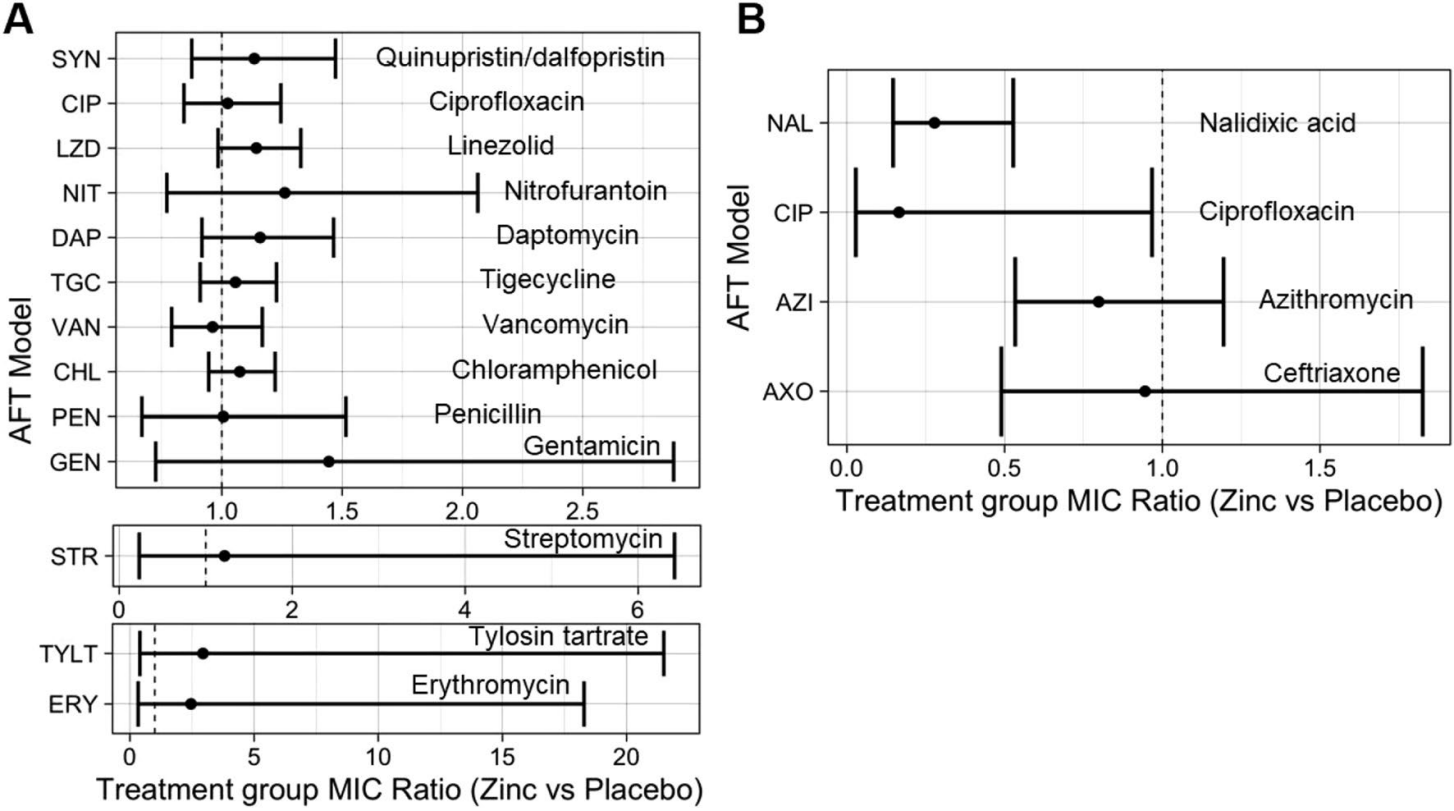
Treatment group minimum inhibitory concentration ratio (MR) estimates and corresponding 95% confidence intervals from accelerated failure time models, after controlling for confounders by (**A**) *Enterococcus* spp. and (**B**) *E. coli*.

For each AFT model, the exponentiated coefficient for the primary effect of treatment group (MIC Ratio, or MR) represents the change in MIC values associated with treatment status, in which a MR > 1 indicates that zinc supplementation was associated with an increase in phenotypic AMR whereas a MR < 1 indicates that zinc supplementation was associated with a decrease in phenotypic AMR^35,36^. Overall findings from AFT models indicated that zinc supplementation was not significantly associated with the MIC of *Enterococcus* spp. to 13 drugs: gentamicin, vancomycin, ciprofloxacin, erythromycin, penicillin, nitrofurantoin, linezolid, quinupristin/dalfopristin, tylosin tartrate, streptomycin, daptomycin, chloramphenicol, and tigecycline (MR = 0.96–2.94, *p* > 0.05) (Fig. 3, Table 2, and Supplementary Tables 10–22). In *E. coli*, no significant association was observed between zinc supplementation and resistance of isolates as measured using MIC values for ceftriaxone (MR = 0.95, *p* > 0.05) and azithromycin (MR = 0.80, *p* > 0.05) with the exception of ciprofloxacin (MR = 0.17, 95% CI 0.03–0.97) and nalidixic acid (MR = 0.28, 95% CI 0.15–0.53) where a protective effect against resistance was observed (Fig. 3, Table 3, and Supplementary Tables 6–9). No significant dose-dependent effect of zinc supplementation was observed across all antimicrobial drugs evaluated for *E. coli* and *Enterococcus* spp..

**Table 2:** Treatment group minimum inhibitory concentration ratio (MR) estimates for *Enterococcus* spp. accelerated failure time models (zinc vs placebo).

Model	Coefficient (SE)	MIC ratio, MR (SE)	*p*-value	95% CI
SYN (quinupristin/dalfopristin)^a^	0.13 (0.13)	1.14 (0.15)	0.340	0.88, 1.47
CIP (ciprofloxacin)^b^	0.024 (0.10)	1.02 (0.10)	0.809	0.84, 1.25
LZD (linezolid)^c^	0.13 (0.076)	1.14 (0.087)	0.079	0.98, 1.33
NIT (nitrofurantoin)^a^	0.23 (0.25)	1.26 (0.32)	0.354	0.77, 2.06
ERY (erythromycin)^b^	0.90 (1.02)	2.46 (2.52)	0.379	0.33, 18.29
TYLT (tylosin tartrate)^b^	1.08 (1.01)	2.94 (2.99)	0.288	0.40, 21.50
DAP (daptomycin)^a^	0.15 (0.12)	1.16 (0.14)	0.216	0.92, 1.46
TGC (tigecycline)^b^	0.055 (0.076)	1.06 (0.081)	0.468	0.91, 1.23
VAN (vancomycin)^a^	− 0.039 (0.099)	0.96 (0.095)	0.694	0.79, 1.17
CHL (chloramphenicol)^b^	0.072 (0.065)	1.07 (0.07)	0.270	0.95, 1.22
PEN (penicillin)^a^	0.0061 (0.21)	1.01 (0.21)	0.977	0.67, 1.52
GEN (gentamicin)^d^	0.37 (0.35)	1.44 (0.51)	0.295	0.73, 2.88
STR (streptomycin)^a^	0.20 (0.85)	1.22 (1.03)	0.816	0.23, 6.42

^a^Model adjusted for age of calf in days and binary spectinomycin treatment variable (received or not).

^b^Model adjusted for age of calf in days and days from last spectinomycin treatment.

^c^Model adjusted for age of calf in days and categorical days from last spectinomycin treatment (0 days, 1–3 days, 4–7 days, and 8–23 days).

^d^Model adjusted for categorical age of calf by week (5–7 days, 8–14 days, 15–21 days, 23–28 days, 29–35 days), and days from last spectinomycin treatment.

**Table 3:** Treatment group minimum inhibitory concentration ratio (MR) estimates for *E. coli* accelerated failure time models (zinc vs placebo).

Model	Coefficient (SE)	MIC ratio, MR (SE)	*p*-value	95% CI
AZI (azithromycin)^a^	− 0.23 (0.21)	0.80 (0.16)	0.273	0.53, 1.19
CIP (ciprofloxacin)^b^	− 1.80 (0.90)	0.17 (0.15)	0.046	0.028, 0.97
NAL (nalidixic acid)^b^	− 1.28 (0.33)	0.28 (0.09)	0.000	0.15, 0.53
AXO (ceftriaxone)^c^	− 0.056 (0.34)	0.95 (0.32)	0.867	0.49, 1.83

^a^Model adjusted for categorical days from last spectinomycin treatment (0 days, 3–5 days, 6–8 days, 9–10 days).

^b^Model adjusted for number of spectinomycin doses received (0, 1, or 2 doses).

^c^Model adjusted for binary spectinomycin treatment variable (received or not) and days on/from diarrhea onset/cure.

### Therapeutic antimicrobial treatment and calf age were significantly associated with AMR

In this study, therapeutic spectinomycin treatment of pre-weaned dairy calves and calf age were significantly associated with AMR for certain antimicrobial drugs in fecal commensal isolates. The effect measures of these confounders represented the corresponding direct effects on AMR when other covariates were held constant; as indirect effects were not accounted for, it should be noted that the magnitude and direction of these associations were not necessarily representative of total effect estimations. Spectinomycin exposure was significantly associated with an increase in MIC for azithromycin, ciprofloxacin, and nalidixic acid in *E. coli* (Supplementary Tables 6, 7, and 8) and tigecycline and linezolid in enterococci isolates (Supplementary Tables 10 and 16). For *Enterococcus* spp., it was also significantly associated with a decrease in MIC for tylosin tartrate, nitrofurantoin, erythromycin, and gentamicin (Supplementary Tables 14, 17, 19, and 22). Calf age at time of sampling was found to be significantly associated with AMR in *Enterococcus* spp. models only; as all *E. coli* isolates were from calves of similar age (14–16 days), age was not evaluated in *E. coli* models. Age was a significant predictor in 5 of the 13 *Enterococcus* spp. models, in which it was significantly associated with a decrease in MIC for tigecycline, nitrofurantoin, penicillin, ciprofloxacin, and gentamicin (Supplementary Tables 10, 17, 18, 20, and 22).

### Predictions of phenotypic antimicrobial resistance

**Figure 4:**
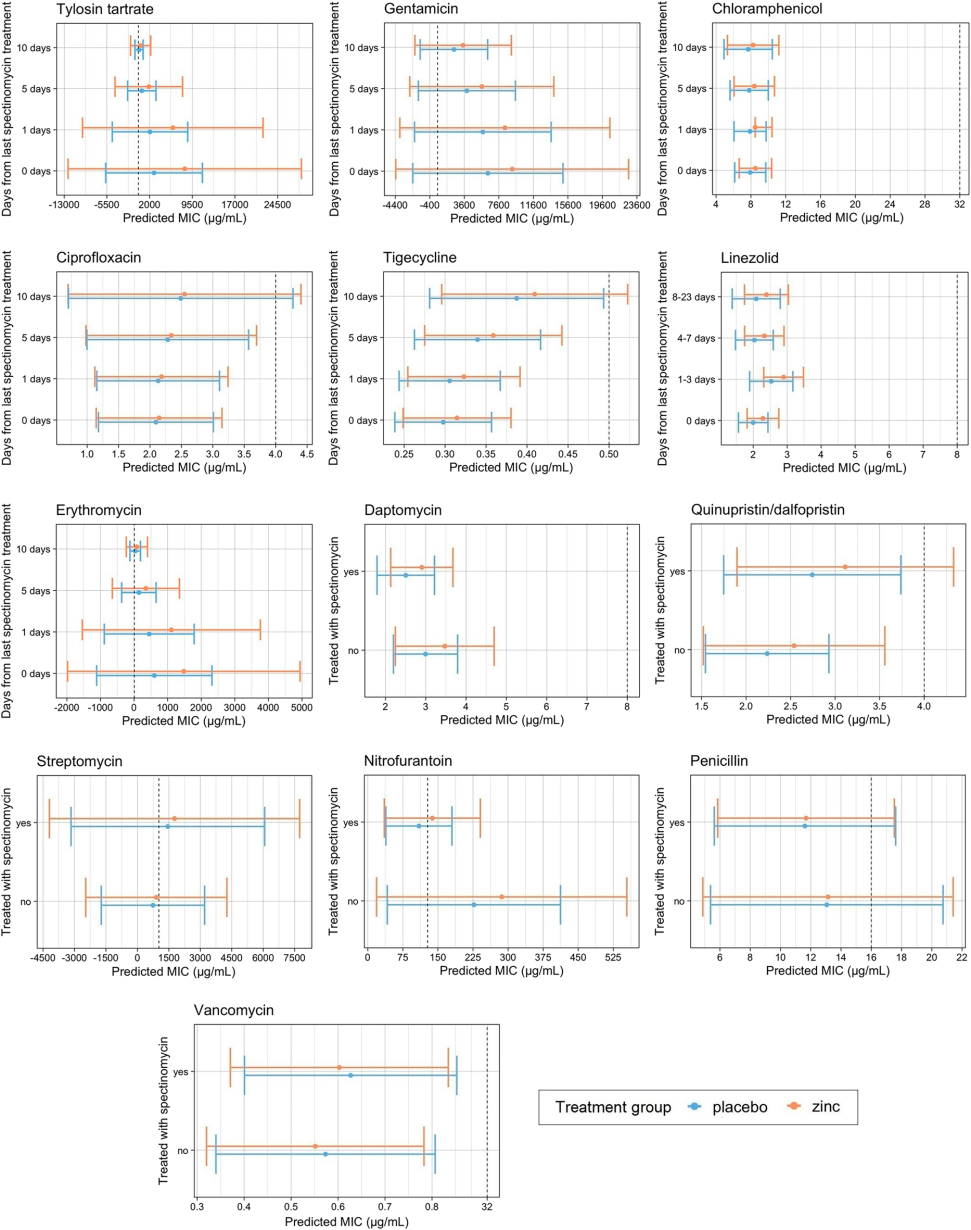
Predicted minimum inhibitory concentrations (MIC, µg/mL) and corresponding 95% confidence intervals for *Enterococcus* spp. accelerated failure time (AFT) models for each antimicrobial drug model and by therapeutic spectinomycin treatment status, controlling for calf age. Dashed lines mark resistant breakpoints based on CLSI criteria for each antimicrobial drug.

**Figure 5:**
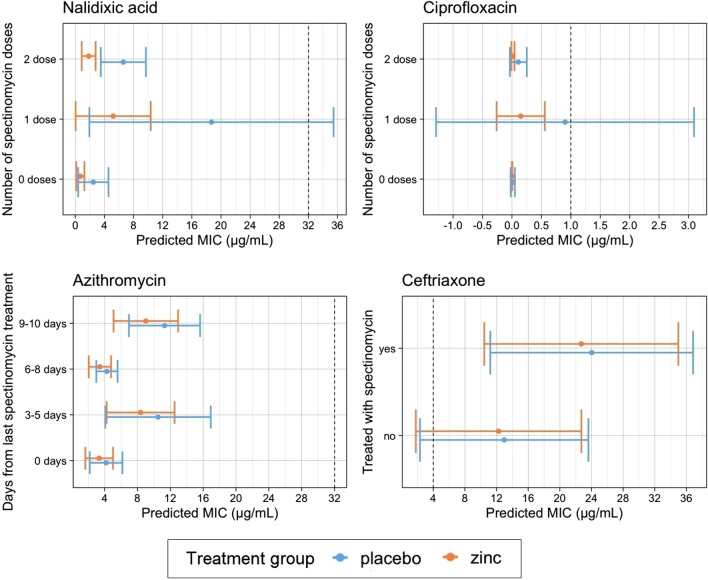
Predicted minimum inhibitory concentration (MIC, µg/mL) and corresponding 95% confidence intervals for *E. coli* accelerated failure time (AFT) models for each antimicrobial drug model, by therapeutic spectinomycin treatment status. Dashed lines mark resistant breakpoints based on CLSI criteria for each antimicrobial drug.

To further understand the dynamics between zinc supplementation and AMR, predictions of MIC from AFT models for *Enterococcus* spp. (Fig. 4) and *E. coli* isolates (Fig. 5) were assessed. The predicted MIC for enterococci isolates after controlling for calf age was higher in zinc-treated compared to placebo-treated calves across all levels of spectinomycin exposure (Fig. 4, Supplementary Tables 27–39). Predicted MIC decreased after spectinomycin exposure for tylosin tartrate, gentamicin, chloramphenicol, erythromycin, daptomycin, nitrofurantoin, and penicillin and increased for ciprofloxacin, tigecycline, quinupristin/dalfopristin, streptomycin, and vancomycin (Fig. 4). In contrast to predictions from *Enterococcus* spp. models, predictions from *E. coli* models indicated that across all levels of spectinomycin exposure, isolates from placebo-treated calves had higher MIC than those from zinc-treated calves for all antimicrobial drugs (Fig. 5, Supplementary Tables 23–26). Differences in MIC were statistically significant between treatment groups for predicted *E. coli* resistance to nalidixic acid for calves that received 2 doses of spectinomycin treatment; *E. coli* from calves in the placebo group that received 2 doses of spectinomycin treatment on average had 4.76 µg/mL higher MIC for nalidixic acid compared to those from calves in the zinc treatment group that received the same number of spectinomycin doses (Fig. 5, Supplementary Table 25). Following spectinomycin exposure, the predicted *E. coli* MIC increased for ceftriaxone (Fig. 5, Supplementary Table 26). For azithromycin, the predicted MIC for *E. coli* exhibited a cyclical trend in days post spectinomycin treatment in which the MIC increased (3–5 days), decreased (6–8 days) and then increased (9–10 days) (Fig. 5, Supplementary Table 23). Across all models, zinc supplementation and therapeutic spectinomycin were not predicted to increase or decrease the MIC to above or below the CLSI resistance breakpoints of tested antimicrobials.

## Discussion

The development of AMR is frequently attributed to use of antimicrobials, but there is increasing recognition that non-antimicrobial agents (e.g. biocides, antiseptics, and heavy metals) may also play a role in the modulation of AMR^37,38^. The large-scale application of these agents in agricultural and livestock production in particular, may pose considerable impact from veterinary medicine, environmental, and public health standpoints; hence, better understanding of non-antimicrobial influences on AMR dynamics in these systems is of great importance. The present study investigated the impact of dietary zinc supplementation in pre-weaned calves on phenotypic AMR using fecal *E. coli* and *Enterococcus* spp. as Gram-negative and Gram-positive indicator organisms, respectively.

In this study, zinc supplementation did not significantly change the MIC to all antimicrobials tested for *E. coli* and *Enterococcus* spp. with the exception of nalidixic acid and ciprofloxacin, in which zinc supplementation was associated with a protective effect against quinolone resistance in *E. coli*. Although the temporality of exposure to zinc preceding resistance cannot be definitively established from our study data due to the cross-sectional nature of evaluating *E. coli* isolates collected at one time point, a previous study evaluating the in vitro selection dynamics of zinc on ciprofloxacin provides evidence in agreement with findings in this study^39^. In laboratory conditions of 0.00625 µg/mL ciprofloxacin and no zinc, ciprofloxacin-resistant *E. coli* was shown to have a significant growth rate advantage relative to a ciprofloxacin-susceptible strain, with this advantage disappearing in the presence of zinc sulfate (0.5 mM and 1.0 mM) at the same ciprofloxacin concentration^39^. Vos et al. further showed through a competition assay that ciprofloxacin-susceptible *E. coli* outcompetes its resistant counterpart under conditions of 0.5 mM zinc sulfate and 0.025 µg/mL ciprofloxacin^39^. While additional work is needed to fully elucidate the role of zinc on quinolone resistance, these congruent findings collectively support the potential antagonistic effects of zinc sulfate on *E. coli* resistance to certain antimicrobial classes.

Limited studies have evaluated the impact of dietary zinc on AMR in calves, with existing zinc supplementation studies conducted in other livestock and evaluating different zinc concentrations. The concentration of zinc fed once per day through milk to pre-weaned dairy calves in this study was 0.22 g zinc sulfate monohydrate, or 80 mg of elemental zinc. Such a non-toxic zinc dose for calves was previously established through a clinical trial^13^, which alongside the field trial that contributed samples for the present analysis and other independent studies, collectively found beneficial impacts of zinc supplementation on the growth performance and diarrheal recovery in pre-weaned calves^14,19,40^. The lack of evidence for the promotion of AMR by dietary zinc in this study is consistent with a factorial trial on feedlot cattle that found copper and/or zinc supplementation (30 or 300 mg/kg) over a 32-day period resulted in minimal changes to MICs of copper, zinc, and antibiotics of fecal *E. coli* and *Enterococcus* spp^25^. These results are further corroborated through another feedlot cattle study, where quantified resistance to ceftriaxone and tetracycline among enteric *E. coli* were unaffected by dietary zinc concentrations (0–90 mg/kg)^26^. In contrast, one feedlot cattle study found that dietary zinc was associated with a significant increase in macrolide resistance of fecal enterococci isolates^27^. The relationship between zinc supplementation and AMR has been more extensively studied in swine, where concentrations of > 2000 mg/kg are typically used in feed additives to prevent post-weaning diarrhea and to enhance growth performance^41^. In contrast to findings from dietary zinc in cattle, multiple studies on swine have found that high dietary zinc may promote AMR, with one study finding that dietary zinc oxide (2,103 mg/kg) increased multi-resistant *E. coli* in feces, digesta, and mucosa^21^; and another finding an increased proportion of multi-resistant *E. coli* in ileum and colon digesta of between zinc oxide (18.6%; 2500 mg/kg) and control (0%; 50 mg/kg) groups^22^. The conflicting findings across these studies suggests that the modulation of AMR by zinc may be in part, or in combination with other unidentified factors, affected by: (1) the host site of bacterial isolation, (2) the duration and dose of supplementation, and (3) the form of zinc (organic/inorganic) administered. In cattle and swine, the absorption of trace minerals such as zinc occurs primarily in the small intestine, with endogenous zinc loss mainly occurring through excretion of feces^42,43^. Differences in the bioavailability and dose of administered zinc may result in variable quantities and duration of zinc presence in the intestinal lumen, which could subsequently exert disparate effects on the gut resistome. Zinc absorption and excretion are homeostatically regulated, with a previous study finding that pigs fed 2000 ppm zinc did not become zinc loaded and excrete increased quantities of zinc in feces until after 13 to 14 days of supplementation^44,45^. Although the digestive tracts of swine (non-ruminant) and cattle (ruminant) at maturity are structurally different, the gastrointestinal (GI) tract of pre-weaned calves is functionally monogastric like that of swine. These GI physiological similarities and the same duration of zinc supplementation evaluated in this study (14 days) suggest that improved health outcomes of pre-weaned calves from zinc supplementation (80 mg elemental zinc) in this study likely occurred with minimal excess zinc excretion.

The age-dependent acquisition and loss of AMR in pre-weaned dairy calves has been well documented to show transient increases in AMR during early calf life^3,5,7,46–49^, which corroborate findings from this study. Higher levels of multiple resistance in fecal *E. coli* isolates were observed in calves 2 weeks of age compared to day-old calves (OR = 53.6)^3^. Additionally, a case–control study evaluating the occurrence of AMR in fecal *E. coli* from diarrheic and healthy dairy calves found higher prevalence of resistance to ampicillin, tetracycline, or sulfonamide (*p* ≤ 0.05) in diarrheic calves^50^. Similarly, this study found high phenotypic AMR (right-censoring of MIC data) in diarrhea-associated *E. coli* isolates collected from calves around 2 weeks of age, which limited our ability to model AMR dynamics of several antimicrobial drugs for *E. coli*. In enterococci isolates, age was significantly associated with a decrease in MIC for 5 of the 13 antimicrobials evaluated after accounting for zinc and spectinomycin exposure. The age-dependent trends in calf AMR has been thought to be driven by host immune status of increased susceptibility during early life that allows ease of colonization by resistant bacteria, exposure through the environment and dam (e.g. colostrum), and rapid establishment of the early gut microbiome^7,8^. A previous study demonstrated that the acquisition of the resistome throughout calf life occurs even in the absence of antimicrobial exposure^8^. Liu et al. also demonstrated that facultative anaerobes such as *Escherichia* and *Enterococcus*, which are frequent carriers of AMR, constitute greater portions of the fecal microbiota during early life but decrease in relative abundance over time^8^. Calf diarrhea has been shown to result in further perturbation of the gut microbiota, with multiple studies observing shifts or differences in gut microbial composition between healthy and diarrheic calves^51–54^. These findings collectively support the hypothesis that stabilization and maturation of the calf gut microbiota with age may impart differential fitness costs for resistance, providing susceptible bacteria a competitive advantage and ultimately leading to a decrease in the resistant enteric microbial population as observed in *Enterococcus* spp. in our study.

Although this study was unable to evaluate the effects of zinc supplementation in absence of antimicrobial exposures, these data provide practical insights on the combined effects of antimicrobial treatment, dietary heavy metal exposure, and host-level factors on phenotypic AMR, interactions that remain undercharacterized. The AMR selective pressures in our study were from dietary milk (same concentration and duration of tetracycline and neomycin for all calves) and therapeutic spectinomycin (same dosage but varied in time and duration of treatment for each calf) which were reflected in the high right-censoring to tested drugs from the same antimicrobial class in our *E. coli* and *Enterococcus* spp. study isolates. These broad-spectrum antibiotics correspond to tetracycline and aminoglycoside/aminocyclitol classes and are commonly used for the treatment of diarrhea in calves; USDA reports from 2014 indicate that of pre-weaned heifers treated with antimicrobials for diarrhea and digestive disorders, aminoglycoside and tetracycline were the third and fourth most commonly administered primary antimicrobials, with 14.7% and 11.2% of calves with these diseases treated with drugs corresponding to these respective classes^12^. The other reported antimicrobials frequently used—third-generation cephalosporins (27.6%) and trimethoprim/sulfamethoxazole (18.7%)^12^—were not administered to calves in our study. Despite this, we also observed right-censored or elevated MICs to these drugs (trimethoprim/sulfamethoxazole and ceftriaxone) in our data, indicating the occurrence of AMR selection in absence of direct selective pressures. Regarding the differential exposure of therapeutic spectinomycin for each calf that was controlled for in all models, neither zinc supplementation nor spectinomycin modulated the predicted MIC of all tested antimicrobials to above or below CLSI resistant breakpoints. These predictions from AFT models allowed for the quantification of absolute changes in resistance to better understand AMR selection dynamics. For example, model predictions indicated that after receiving two doses of therapeutic spectinomycin, *E. coli* from placebo-treated calves had a significantly higher nalidixic acid MIC relative to those from zinc-treated calves. These predictions support aforementioned in vivo findings that the putative inhibitory effects of zinc on quinolone resistance may occur in combination with antimicrobial selective pressures^39^. Antimicrobial therapy has been shown to transiently modify the calf gut microbiota, which may favorably enrich resistant bacteria^55,56^. Indeed, model predictions from this study indicated that following spectinomycin treatment, MICs to certain antimicrobials increase in both *E. coli* (ceftriaxone) and *Enterococcus* spp. (ciprofloxacin, tigecycline, quinupristin/dalfopristin, streptomycin, and vancomycin). However, the decreasing trend in MIC to other antimicrobials—primarily in *Enterococcus* spp. to tylosin tartrate, gentamicin, chloramphenicol, erythromycin, daptomycin, nitrofurantoin, and penicillin—and the cyclical pattern in azithromycin MIC of *E. coli* following spectinomycin treatment underscores the complexity of AMR selection. With respect to the latter finding, previous studies have observed similar cyclical resistance trends in fecal *Enterobacteriaceae* from dairy cattle following antimicrobial treatment. This included one study that observed cyclical peaks in neomycin resistant *Enterobacteriaceae* counts following treatment with the drug^57^, and another which observed cyclical re-emergence of ceftiofur-resistant *Enterobacteriaceae* counts following systemic antimicrobial drug treatment^58^. In some bacteria, antimicrobial exposure has been shown to induce mutagenesis and conjugation^59–61^, which can be followed by emergence of additional compensatory mutations that alleviate fitness costs associated with resistance (e.g. positive epistasis)^62,63^. Such variation in fitness costs would support how modifications of the calf enteric microbiota from collective influences of antimicrobial therapy, disease (e.g. diarrhea), and other changes in host-factors culminate a microbial system that is conducive to the divergent and bidirectional selection of bacterial resistance.

In conclusion, the current study found a lack of evidence for the selection of phenotypic AMR to the majority of tested antimicrobial drugs in fecal commensal bacteria from dietary zinc supplementation in pre-weaned dairy calves. The primary limitations of this study include the cross-sectional assessment of *E. coli* at one time point which limited our ability to determine the temporality of association between dietary zinc treatment and AMR, and the small sample size of *E. coli* which may impact the reliability of study findings. Additionally, our study may have limited external validity due to samples originating from just one dairy. Future studies conducted across different dairies with varied environmental factors and management practices, with larger sample sizes, and evaluating multiple isolates per sample are needed to validate the effect sizes observed in our study, particularly for the significant findings of the antagonistic association between zinc supplementation and quinolone resistance in *E. coli*. As our main objective was to assess the impact of dietary zinc on AMR, AFT models specified in this study were not designed to obtain unbiased point estimates on the relationship of calf age and antimicrobial treatment with AMR. Additionally, the extreme model estimates observed in our study for two models (gentamicin and streptomycin for *Enterococcus* spp.) indicated that AFT models may not be optimal for data with a high frequency of right- and left- censoring; hence, alternative approaches such as truncated interval censored models should be explored for these types of data in future studies. Further research that evaluates a longer follow-up period (e.g. through post-weaning), the impact of other forms of dietary zinc (e.g. chelated zinc), and different resistance outcomes are needed. Additionally, genomic analysis of isolates in this study would provide additional insight on the AMR dynamics of dietary zinc supplementation, antimicrobial treatment, and disease status in pre-weaned dairy calves. Collectively, this work corroborates previous reports of host-level drivers (e.g. age and antimicrobial exposure) on AMR, and provides important insights into the largely unexplored dynamics of dietary zinc and AMR in pre-weaned dairy calves.

### Supplementary Information

Supplementary Information.

## Data Availability

Data from this study are presented in the Supplementary File. Additional source data are available from the corresponding author upon reasonable request.

## References

[1] Murray CJ (2022). Global burden of bacterial antimicrobial resistance in 2019: A systematic analysis. Lancet.

[2] Economou V, Gousia P (2015). Agriculture and food animals as a source of antimicrobial-resistant bacteria. Infect. Drug Resist..

[3] Berge ACB, Atwill ER, Sischo WM (2005). Animal and farm influences on the dynamics of antibiotic resistance in faecal *Escherichia coli* in young dairy calves. Prev. Vet. Med..

[4] Berge ACB, Moore DA, Sischo WM (2006). Field trial evaluating the influence of prophylactic and therapeutic antimicrobial administration on antimicrobial resistance of fecal *Escherichia coli* in dairy calves. Appl. Environ. Microbiol..

[5] Li X (2018). Phenotypic antimicrobial resistance profiles of *E. coli* and *Enterococcus* from dairy cattle in different management units on a Central California dairy. Clin. Microbiol..

[6] Oh S-I, Ha S, Roh J-H, Hur T-Y, Yoo JG (2020). Dynamic changes in antimicrobial resistance in fecal *Escherichia coli* from neonatal dairy calves: An individual follow-up study. Animals.

[7] Khachatryan AR, Hancock DD, Besser TE, Call DR (2004). Role of calf-adapted *Escherichia coli* in maintenance of antimicrobial drug resistance in dairy calves. Appl. Environ. Microbiol..

[8] Liu J (2019). The fecal resistome of dairy cattle is associated with diet during nursing. Nat. Commun..

[9] Cho Y, Yoon K-J (2014). An overview of calf diarrhea—infectious etiology, diagnosis, and intervention. J. Vet. Sci..

[10] Constable PD (2004). Antimicrobial use in the treatment of calf diarrhea. J. Vet. Intern. Med..

[11] Constable PD (2009). Treatment of calf diarrhea: Antimicrobial and ancillary treatments. Vet. Clin. N. Am. Food Anim. Pract..

[12] USDA. *Health and Management Practices on U.S. Dairy Operations, 2014*; https://www.aphis.usda.gov/animal_health/nahms/dairy/downloads/dairy14/Dairy14_dr_PartIII.pdf. Accessed 20 October 2023.

[13] Glover AD (2013). A double-blind block randomized clinical trial on the effect of zinc as a treatment for diarrhea in neonatal Holstein calves under natural challenge conditions. Prev. Vet. Med..

[14] Feldmann HR, Williams DR, Champagne JD, Lehenbauer TW, Aly SS (2019). Effectiveness of zinc supplementation on diarrhea and average daily gain in pre-weaned dairy calves: A double-blind, block-randomized, placebo-controlled clinical trial. PLOS One.

[15] Bonaventura P, Benedetti G, Albarède F, Miossec P (2015). Zinc and its role in immunity and inflammation. Autoimmun. Rev..

[16] Maywald M, Wessels I, Rink L (2017). Zinc signals and immunity. Int. J. Mol. Sci..

[17] Ma FT (2020). Zinc-methionine acts as an anti-diarrheal agent by protecting the intestinal epithelial barrier in postnatal Holstein dairy calves. Anim. Feed Sci. Technol..

[18] Liu J, Ma F, Degen A, Sun P (2023). The effects of zinc supplementation on growth, diarrhea, antioxidant capacity, and immune function in Holstein dairy calves. Animals.

[19] Chang MN (2020). Effects of different types of zinc supplement on the growth, incidence of diarrhea, immune function, and rectal microbiota of newborn dairy calves. J. Dairy Sci..

[20] McNeilly O, Mann R, Hamidian M, Gunawan C (2021). Emerging concern for silver nanoparticle resistance in *Acinetobacter baumannii* and other bacteria. Front. Microbiol..

[21] Ciesinski L (2018). High dietary zinc feeding promotes persistence of multi-resistant *E. coli* in the swine gut. PLoS One.

[22] Bednorz C (2013). The broader context of antibiotic resistance: Zinc feed supplementation of piglets increases the proportion of multi-resistant *Escherichia coli* in vivo. Int. J. Med. Microbiol..

[23] Johanns VC (2019). Effects of a four-week high-dosage zinc oxide supplemented diet on commensal *Escherichia coli* of weaned pigs. Front. Microbiol..

[24] Vahjen W, Pietruszyńska D, Starke IC, Zentek J (2015). High dietary zinc supplementation increases the occurrence of tetracycline and sulfonamide resistance genes in the intestine of weaned pigs. Gut Pathog..

[25] Jacob ME (2010). Effects of feeding elevated concentrations of copper and zinc on the antimicrobial susceptibilities of fecal bacteria in feedlot cattle. Foodborne Pathog. Dis..

[26] Van Bibber-Krueger CL (2019). Effects of supplemental zinc sulfate on growth performance, carcass characteristics, and antimicrobial resistance in feedlot heifers1. J. Anim. Sci..

[27] Murray SA (2021). Effects of zinc and menthol-based diets on co-selection of antibiotic resistance among *E. coli* and *Enterococcus* spp. in beef cattle. Animals.

[28] Atwill ER (2015). Transfer of *Escherichia coli* O157:H7 from simulated wildlife scat onto romaine lettuce during foliar irrigation. J. Food Prot..

[29] Anastasi EM (2010). Prevalence and persistence of *Escherichia coli* strains with uropathogenic virulence characteristics in sewage treatment plants. Appl. Environ. Microbiol..

[30] Chen J, Griffiths MW (1998). PCR differentiation of *Escherichia coli* from other Gram-negative bacteria using primers derived from the nucleotide sequences flanking the gene encoding the universal stress protein. Lett. Appl. Microbiol..

[31] Ludwig W, Schleifer K-H (2000). How quantitative is quantitative PCR with respect to cell counts?. Syst. Appl. Microbiol..

[32] Frahm E, Obst U (2003). Application of the fluorogenic probe technique (TaqMan PCR) to the detection of *Enterococcus* spp. and *Escherichia coli* in water samples. J. Microbiol. Methods.

[33] STATA. https://www.stata.com/manuals/rvce_option.pdf#rvce_option. Accessed 20 October 2023.

[34] STATA. https://www.stata.com/manuals/u20.pdf#u20.22Obtainingrobustvarianceestimates. Accessed 20 October 2023.

[35] Breen MJ (2023). Effect of group housing of preweaned dairy calves: Health and fecal commensal antimicrobial resistance outcomes. Antibiotics.

[36] Okello E, ElAshmawy WR, Williams DR, Lehenbauer TW, Aly SS (2023). Effect of dry cow therapy on antimicrobial resistance of mastitis pathogens post-calving. Front. Vet. Sci..

[37] Poole K (2017). At the nexus of antibiotics and metals: The impact of Cu and Zn on antibiotic activity and resistance. Trends Microbiol..

[38] Wales AD, Davies RH (2015). Co-selection of resistance to antibiotics, biocides and heavy metals, and its relevance to foodborne pathogens. Antibiotics.

[39] Vos M (2020). Zinc can counteract selection for ciprofloxacin resistance. FEMS Microbiol. Lett..

[40] Wo Y (2022). Supplementation with zinc proteinate increases the growth performance by reducing the incidence of diarrhea and improving the immune function of dairy calves during the first month of life. Front. Vet. Sci..

[41] Burrough ER, De Mille C, Gabler NK (2019). Zinc overload in weaned pigs: Tissue accumulation, pathology, and growth impacts. J. Vet. Diagn. Invest..

[42] Broom LJ, Monteiro A, Piñon A (2021). Recent advances in understanding the influence of zinc, copper, and manganese on the gastrointestinal environment of pigs and poultry. Animals.

[43] Miller WJ (1970). Zinc nutrition of cattle: A review. J. Dairy Sci..

[44] Rincker MJ, Hill GM, Link JE, Meyer AM, Rowntree JE (2005). Effects of dietary zinc and iron supplementation on mineral excretion, body composition, and mineral status of nursery pigs1,2. J. Anim. Sci..

[45] Hill GM, Shannon MC (2019). Copper and zinc nutritional issues for agricultural animal production. Biol. Trace Elem. Res..

[46] Cao H (2019). Age-associated distribution of antimicrobial-resistant *Salmonella enterica* and *Escherichia coli* Isolated from dairy herds in Pennsylvania, 2013–2015. Foodborne Pathog. Dis..

[47] Carey AM (2022). Prevalence and profiles of antibiotic resistance genes mph(A) and qnrB in extended-spectrum beta-lactamase (ESBL)-producing *Escherichia coli* isolated from dairy calf feces. Microorganisms.

[48] Gaire TN, Scott HM, Sellers L, Nagaraja TG, Volkova VV (2021). Age dependence of antimicrobial resistance among fecal bacteria in animals: A scoping review. Front. Vet. Sci..

[49] Merle R (2023). The therapy frequency of antibiotics and phenotypical resistance of *Escherichia coli* in calf rearing sites in Germany. Front. Vet. Sci..

[50] de Verdier K, Nyman A, Greko C, Bengtsson B (2012). Antimicrobial resistance and virulence factors in *Escherichia coli* from Swedish dairy calves. Acta Vet. Scand..

[51] Gomez DE, Arroyo LG, Costa MC, Viel L, Weese JS (2017). Characterization of the fecal bacterial microbiota of healthy and diarrheic dairy calves. J. Vet. Intern. Med..

[52] Fan P (2021). The gut microbiota of newborn calves and influence of potential probiotics on reducing diarrheic disease by inhibition of pathogen colonization. Front. Microbiol..

[53] Kim E-T (2021). Dynamic changes in fecal microbial communities of neonatal dairy calves by aging and diarrhea. Animals.

[54] Malmuthuge N, Guan LL (2017). Understanding the gut microbiome of dairy calves: Opportunities to improve early-life gut health. J. Dairy Sci..

[55] Beyi AF (2021). Enrofloxacin alters fecal microbiota and resistome irrespective of its dose in calves. Microorganisms.

[56] Ma T (2020). Linking perturbations to temporal changes in diversity, stability, and compositions of neonatal calf gut microbiota: Prediction of diarrhea. ISME J..

[57] Cella E (2021). Estimating the rates of acquisition and loss of resistance of enterobacteriaceae to antimicrobial drugs in pre-weaned dairy calves. Microorganisms.

[58] Sheedy DB (2021). Effect of antimicrobial treatment on the dynamics of ceftiofur resistance in enterobacteriaceae from adult california dairy cows. Microorganisms.

[59] Lopatkin AJ (2016). Antibiotics as a selective driver for conjugation dynamics. Nat. Microbiol..

[60] Revitt-Mills SA, Robinson A (2020). Antibiotic-Induced Mutagenesis: Under the microscope. Front. Microbiol..

[61] Ding M (2022). Subinhibitory antibiotic concentrations promote the horizontal transfer of plasmid-borne resistance genes from *Klebsiellae pneumoniae* to *Escherichia coli*. Front. Microbiol..

[62] Marcusson LL, Frimodt-Møller N, Hughes D (2009). Interplay in the selection of fluoroquinolone resistance and bacterial fitness. PLOS Pathog..

[63] Melnyk AH, Wong A, Kassen R (2015). The fitness costs of antibiotic resistance mutations. Evolut. Appl..

