# Supplementary material for: Impact of zinc supplementation on phenotypic antimicrobial resistance of fecal commensal bacteria from pre-weaned dairy calves
Source: Sci Rep. 2024 Feb 23;14:4448. doi: 10.1038/s41598-024-54738-x (PMC10891156; doi:10.1038/s41598-024-54738-x)
Supplement: Supplementary file 1 — Supplementary Information. [file 41598_2024_54738_MOESM1_ESM.pdf]

Supplementary Table 1. Primer sequences used for PCR confirmation of *E. coli* and *Enterococcus* spp.

Organism	Primer sequence	
E. coli	Forward	5'-CCG ATA CGC TGC CAA TCA GT-3'
E. coli	Reverse	5'-ACG CAG ACC GTA GGC CAG AT-3'
Enterococcus spp.	Forward	5'-AGA AAT TCC AAA CGA ACT TG-3'
Enterococcus spp.	Reverse	5'-CAG TGC TCT ACC TCC ATC ATT-3'

Supplementary Table 2. Drug panel and dilutions used for antimicrobial susceptibility testing of *E. coli* isolates

Drug Class	Abbreviation	Antimicrobial drug	Range (µg/mL)
beta-lactam	FOX	Cefoxitin	0.5 - 32
macrolide	AZI	Azithromycin	0.12 - 16
chloramphenicol	CHL	Chloramphenicol	2 - 32
tetracycline	TET	Tetracycline	4 - 32
beta-lactam	AXO	Ceftriaxone	0.25 - 64
beta-lactam	AUG2	Amoxicillin/clavulanic acid	1/0.5 - 32/16
quinolone	CIP	Ciprofloxacin	0.015 - 4
aminoglycoside	GEN	Gentamicin	0.25 - 16
quinolone	NAL	Nalidixic acid	0.5 - 32
beta-lactam	XNL	Ceftiofur	0.12 - 8
sulfonamide	FIS	Sulfisoxazole	16 - 256
sulfonamide	SXT	Trimethoprim-sulfamethoxazole	0.12/2.38 - 4/76
beta-lactam	AMP	Ampicillin	1-32
aminoglycoside	STR	Streptomycin	2.64

Supplementary Table 3. Drug panel and dilutions used for antimicrobial susceptibility testing of *Enterococcus* spp. isolates

Drug Class	Abbreviation	Antimicrobial drug	Range (µg/mL)
glycylcycline	TCG	Tigecycline	0.015 - 0.5
tetracycline	TET	Tetracycline	1-32
chloramphenicol	CHL	Chloramphenicol	2-32
lipopeptide	DAP	Daptomycin	0.25 - 16
aminoglycoside	STR	Streptomycin	512 - 2048
macrolide	TYLT	Tylosin tartrate	0.25 - 32
streptogramin	SYN	Quinupristin / dalfopristin	0.5 - 32
oxazolidinone	LZD	Linezolid	0.5 - 8
nitrofurans	NIT	Nitrofurantoin	2-64
beta-lactam	PEN	Penicillin	0.25 - 16
aminoglycoside	KAN	Kanamycin	128 - 1024
macrolide	ERY	Erythromycin	0.25 - 8
quinolone	CIP	Ciprofloxacin	0.12 - 4
glycopeptide	VAN	Vancomycin	0.25 - 32
lincosamide	LIN	Lincomycin	1-8
aminoglycoside	GEN	Gentamicin	128 - 1024

Supplementary Figure 1. Distribution of minimum inhibitory concentration from antimicrobial susceptibility testing for all *E. coli* isolates (n=44). Shaded areas indicate the range of tested antimicrobials. Numbers listed outside of the shaded range correspond to right/left censored counts.

	Distribution of MIC (μg/mL) - Number of *E. coli* isolates (n=44)																
Antimicrobial drug (Abbreviation)		0.015	0.03	0.06	0.12	0.25	0.5	1	2	4	8	16	32	64	128	256	
Cefoxitin (FOX)							0	0	0	2	0	1	12	29			
Azithromycin (AZI)					0	0	0	0	2	23	13	2	4				
Chloramphenicol (CHL)									0	0	9	2	0	33			
Tetracycline (TET)										0	0	0	0	44			
Ceftriaxone (AXO)					2	0	0	0	0	0	12	17	8	2	3		
Amoxicillin/clavulanic acid (AUG2)								0	0	0	3	0	37	4			
Ciprofloxacin (CIP)	16	0	21	1	0	0	2	0	0	0	4						
Gentamicin (GEN)						0	2	3	1	0	0	0	38				
Nalidixic acid (NAL)							0	0	30	9	0	1	0	4			
Ceftiofur (XNL)					0	0	2	0	0	1	17	24					
Sulfisoxazole (FIS)												0	0	0	0		44
Trimethoprim-sulfamethoxazole (SXT)					0	0	0	0	0	0	44						
Ampicillin (AMP)								0	0	0	0	0	0	44			
Streptomycin (STR)									0	0	0	1	0	0	43		

Supplementary Figure 2. Distribution of minimum inhibitory concentration from antimicrobial susceptibility testing for *E. coli* isolates from placebo calves (n=26). Shaded areas indicate the range of tested antimicrobials. Numbers listed outside of the shaded range correspond to right/left censored counts.

	Distribution of MIC (μg/mL) - Number of *E. coli* isolates (n=26)																
Antimicrobial drug (Abbreviation)		0.015	0.03	0.06	0.12	0.25	0.5	1	2	4	8	16	32	64	128	256	
Cefoxitin (FOX)							0	0	0	1	0	0	6	19			
Azithromycin (AZI)					0	0	0	0	2	13	8	0	3				
Chloramphenicol (CHL)									0	0	3	2	0	21			
Tetracycline (TET)										0	0	0	0	26			
Ceftriaxone (AXO)					1	0	0	0	0	0	7	10	5	2	1		
Amoxicillin/clavulanic acid (AUG2)								0	0	0	1	0	22	3			
Ciprofloxacin (CIP)	10	0	10	1	0	0	1	0	0	0	4						
Gentamicin (GEN)						0	1	3	1	0	0	0	21				
Nalidixic acid (NAL)							0	0	17	4	0	1	0	4			
Ceftiofur (XNL)					0	0	1	0	0	1	9	15					
Sulfisoxazole (FIS)												0	0	0	0	0	26
Trimethoprim-sulfamethoxazole (SXT)					0	0	0	0	0	0	26						
Ampicillin (AMP)								0	0	0	0	0	0	26			
Streptomycin (STR)									0	0	0	1	0	0	25		

Supplementary Figure 3. Distribution of minimum inhibitory concentration from antimicrobial susceptibility testing for *E. coli* isolates from zinc treatment calves (n=18). Shaded areas indicate the range of tested antimicrobials. Numbers listed outside of the shaded range correspond to right/left censored counts.

	Distribution of MIC (μg/mL) - Number of *E. coli* isolates (n=18)																
Antimicrobial drug (Abbreviation)		0.015	0.03	0.06	0.12	0.25	0.5	1	2	4	8	16	32	64	128	256	
Cefoxitin (FOX)							0	0	0	1	0	1	6	10			
Azithromycin (AZI)					0	0	0	0	0	10	5	2	1				
Chloramphenicol (CHL)									0	0	6	0	0	12			
Tetracycline (TET)										0	0	0	0	18			
Ceftriaxone (AXO)					1	0	0	0	0	0	5	7	3	0	2		
Amoxicillin/clavulanic acid (AUG2)								0	0	0	2	0	15	1			
Ciprofloxacin (CIP)	6	0	11	0	0	0	1	0	0	0							
Gentamicin (GEN)						0	1	0	0	0	0	0	17				
Nalidixic acid (NAL)							0	0	13	5	0	0	0				
Ceftiofur (XNL)					0	0	1	0	0	0	8	9					
Sulfisoxazole (FIS)												0	0	0	0	0	18
Trimethoprim-sulfamethoxazole (SXT)					0	0	0	0	0	0	18						
Ampicillin (AMP)								0	0	0	0	0	0	18			
Streptomycin (STR)									0	0	0	0	0	0	18		

Supplementary Table 4. Descriptive statistics for minimum inhibitory concentrations from antimicrobial susceptibility testing for *E. coli* isolates.

**Antimicrobial drug**	**All isolates**		**Placebo isolates**		**Zinc isolates**	
	MIC_{50}	MIC_{90}	MIC_{50}	MIC_{90}	MIC_{50}	MIC_{90}
Cefoxitin (FOX)	>32	>32	>32	>32	>32	>32
Azithromycin (AZI)	4	16	4	>16	4	16
Chloramphenicol (CHL)	>32	>32	>32	>32	>32	>32
Tetracycline (TET)	>32	>32	>32	>32	>32	>32
Ceftriaxone (AXO)	16	64	16	64	16	>64
Amoxicillin/clavulanic acid (AUG2)	32	32	32	>32	32	32
Ciprofloxacin (CIP)	0.03	0.5	0.03	>4	0.03	0.03
Gentamicin (GEN)	>16	>16	>16	>16	>16	>16
Nalidixic acid (NAL)	2	16	2	>32	2	4
Ceftiofur (XNL)	>8	>8	>8	>8	8	>8
Sulfisoxazole (FIS)	>128	>128	>128	>128	>128	>128
Trimethoprim-sulfamethoxazole (SXT)	>4	>4	>4	>4	>4	>4
Ampicillin (AMP)	>32	>32	>32	>32	>32	>32
Streptomycin (STR)	>64	>64	>64	>64	>64	>64

Supplementary Figure 4. Distribution of minimum inhibitory concentration from antimicrobial susceptibility testing for all *Enterococcus* spp. isolates (n=167). Shaded areas indicate the range of tested antimicrobials. Numbers listed outside of the shaded range correspond to right/left censored counts.

Antimicrobial drug	Distribution of MIC (µg/mL) - Number of *Enterococcus* spp. isolates (n=167)																			
	0.012	0.015	0.03	0.06	0.12	0.25	0.5	1	2	4	8	16	32	64	128	256	512	1024	2048	4096
Tigecycline		0	0	7	39	75	46													
Tetracycline								0	0	0	0	0	1	166						
Chloramphenicol									0	0	53	91	3	20						
Daptomycin						0	12	45	22	80	7	1								
Streptomycin																23	0	8	4	132
Tylosin tartrate						0	1	9	17	12	0	1	0	127						
Quinupristin / dalfopristin						4	0	18	36	85	5	17	1	1						
Linezolid							0	0	75	89	3									
Nitrofurantoin									0	0	29	37	3	28	70					
Penicillin						0	0	10	63	17	44	13	20							
Kanamycin														2	0	0	0	0	165	
Erythromycin					4	0	25	0	5	4	1	128								
Ciprofloxacin					0	0	0	7	60	48	52									
Vancomycin						0	57	58	50	1	0	0	1							
Lincomycin							4	0	0	0	2	161								
Gentamicin														80	0	1	0	0	86	

Supplementary Figure 5. Distribution of minimum inhibitory concentration from antimicrobial susceptibility testing for *Enterococcus* spp. isolates from placebo calves (n=100). Shaded areas indicate the range of tested antimicrobials. Numbers listed outside of the shaded range correspond to right/left censored counts.

Antimicrobial drug	Distribution of MIC (µg/mL) - Number of *Enterococcus* spp. isolates (n=100)																			
	0.012	0.015	0.03	0.06	0.12	0.25	0.5	1	2	4	8	16	32	64	128	256	512	1024	2048	4096
Tigecycline		0	0	2	24	50	24													
Tetracycline								0	0	0	0	0	1	99						
Chloramphenicol									0	0	35	53	1	11						
Daptomycin						0	6	29	13	49	3	0								
Streptomycin																14	0	4	2	80
Tylosin tartrate						0	1	7	10	7	0	1	0	74						
Quinupristin / dalfopristin						3	0	12	22	50	3	9	0	1						
Linezolid							0	0	50	49	1									
Nitrofurantoin									0	0	20	20	3	16	41					
Penicillin						0	0	7	38	8	27	7	13							
Kanamycin														2	0	0	0	0	98	
Erythromycin					2	0	17	0	3	3	0	75								
Ciprofloxacin					0	0	0	4	38	28	30									
Vancomycin						0	33	34	33	0	0	0	0							
Lincomycin							4	0	0	0	1	95								
Gentamicin														52	0	1	0	0	47	

Supplementary Figure 6. Distribution of minimum inhibitory concentration from antimicrobial susceptibility testing for *Enterococcus* spp. isolates from zinc treatment calves (n=67). Shaded areas indicate the range of tested antimicrobials. Numbers listed outside of the shaded range correspond to right/left censored counts.

Antimicrobial drug	Distribution of MIC (µg/mL) - Number of *Enterococcus* spp. isolates (n=67)																			
	0.012	0.015	0.03	0.06	0.12	0.25	0.5	1	2	4	8	16	32	64	128	256	512	1024	2048	4096
Tigecycline		0	0	5	15	25	22													
Tetracycline								0	0	0	0	0	0	67						
Chloramphenicol									0	0	18	38	2	9						
Daptomycin						0	6	16	9	31	4	1								
Streptomycin																9	0	4	2	52
Tylosin tartrate						0	0	2	7	5	0	0	0	53						
Quinupristin / dalfopristin						1	0	6	14	35	2	8	1							
Linezolid							0	0	25	40	2									
Nitrofurantoin									0	0	9	17	0	12	29					
Penicillin						0	0	3	25	9	17	6	7							
Kanamycin															0	0	0	0	67	
Erythromycin					2	0	8	0	2	1	1	53								
Ciprofloxacin					0	0	0	3	22	20	22									
Vancomycin						0	24	24	17	1	0	0	1							
Lincomycin								0	0	0	1	67								
Gentamicin														28	0	0	0	0	39	

Supplementary Table 5. Descriptive statistics for minimum inhibitory concentrations from antimicrobial susceptibility testing for *Enterococcus* isolates.

**Antimicrobial drug**	**All isolates**		**Placebo isolates**		**Zinc isolates**	
	MIC_{50}	MIC_{90}	MIC_{50}	MIC_{90}	MIC_{50}	MIC_{90}
Tigecycline (TGC)	0.25	0.5	0.25	0.5	0.25	0.5
Tetracycline (TET)	>32	>32	>32	>32	>32	>32
Chloramphenicol (CHL)	16	>32	16	>32	16	>32
Daptomycin (DAP)	4	4	4	4	4	4
Streptomycin (STR)	>2048	>2048	>2048	>2048	>2048	>2048
Tylosin tartrate (TYLT)	>32	>32	>32	>32	>32	>32
Quinupristin/dalfopristin (SYN)	4	16	4	8	4	16
Linezolid (LZD)	4	4	2	4	4	4
Nitrofurantoin (NIT)	64	>64	64	>64	64	>64
Penicillin (PEN)	4	>16	4	>16	4	>16
Kanamycin (KAN)	>1024	>1024	>1024	>1024	>1024	>1024
Erythromycin (ERY)	>8	>8	>8	>8	>8	>8
Ciprofloxacin (CIP)	4	>4	4	>4	4	>4
Vancomycin (VAN)	1	2	1	2	1	2
Lincomycin (LIN)	>8	>8	>8	>8	>8	>8
Gentamicin (GEN)	>1024	>1024	<=128	>1024	>1024	>1024

Supplementary Table 6. Final accelerated failure time (AFT) model for azithromycin minimum inhibitory concentrations for *E. coli* isolates (BIC 144.0987, Weibull distribution).

Factor	Level	Coefficient (SE)	MIC Ratio (SE)	P-value	95% CI
Treatment group	Zinc	-0.23 (0.21)	0.80 (0.16)	0.273	0.53, 1.19
	Placebo	Referent	-	-	-
Days from last spectinomycin treatment	3-5 days	0.92 (0.35)	2.51 (0.89)	0.009	1.26, 5.02
	6-8 days	0.025 (0.27)	1.03 (0.28)	0.926	0.60, 1.74
	9-10 days	1.00 (0.29)	2.70 (0.79)	0.001	1.53, 4.78
	0 days	Referent	-	-	-
Intercept	-	1.43 (0.24)	4.18 (1.02)	0	2.59, 6.73

Supplementary Table 7. Final accelerated failure time (AFT) model for ciprofloxacin minimum inhibitory concentrations for *E. coli* isolates (BIC 189.5612, Weibull distribution).

Factor	Level	Coefficient (SE)	MIC Ratio (SE)	P-value	95% CI
Treatment group	Zinc	-1.80 (0.90)	0.17 (0.15)	0.046	0.028, 0.97
	Placebo	Referent	-	-	-
Number of spectinomycin doses received	1	4.12 (1.67)	61.64 (102.96)	0.014	2.33, 1627.81
	2	2.01 (1.20)	7.45 (8.92)	0.094	0.71, 77.96
	0	Referent	-	-	-
Intercept	-	-4.22 (1.24)	0.015 (0.018)	0.001	0.0013, 0.17

Supplementary Table 8. Final accelerated failure time (AFT) model for nalidixic acid minimum inhibitory concentrations for *E. coli* isolates (BIC 181.116, Exponential distribution).

Factor	Level	Coefficient (SE)	MIC Ratio (SE)	P-value	95% CI
Treatment group	Zinc	-1.28 (0.33)	0.28 (0.09)	0	0.15, 0.53
	Placebo	Referent	-	-	-
Number of spectinomycin doses received	1	2.02 (0.60)	7.57 (4.55)	0.001	2.33, 24.57
	2	0.98 (0.44)	2.67 (1.16)	0.024	1.14, 6.27
	0	Referent	-	-	-
Intercept	-	0.90 (0.43)	2.47 (1.06)	0.036	1.06, 5.73

Supplementary Table 9. Final accelerated failure time (AFT) model for ceftriaxone minimum inhibitory concentrations for *E. coli* isolates (BIC 172.8303, Exponential distribution).

Factor	Level	Coefficient (SE)	MIC Ratio (SE)	P-value	95% CI
Treatment group	Zinc	-0.056 (0.34)	0.95 (0.32)	0.867	0.49, 1.83
	Placebo	Referent	-	-	-
Days on/from diarrhea		0.095 (0.049)	1.10 (0.054)	0.052	1.00, 1.21
Received spectinomycin treatment	Yes	0.62 (0.43)	1.85 (0.80)	0.152	0.80, 4.31
	No	Referent	-	-	-
Intercept	-	2.56 (0.42)	12.97 (5.43)	0	5.71, 29.47

Supplementary Table 10. Final accelerated failure time (AFT) model for tigecycline minimum inhibitory concentrations for enterococci isolates (BIC 422.7243, Weibull distribution).

Factor	Level	Coefficient (SE)	MIC Ratio (SE)	P-value	95% CI
Treatment group	Zinc	0.055 (0.076)	1.06 (0.081)	0.468	0.91, 1.23
	Placebo	Referent	-	-	-
Age of calf (days)	-	-0.031 (0.0065)	0.97 (0.0063)	0	0.96, 0.98
Days from spectinomycin treatment	-	0.026 (0.0078)	1.03 (0.0080)	0.001	1.01, 1.04
Intercept	-	-1.21 (0.10)	0.30 (0.030)	0	0.24, 0.36

Supplementary Table 11. Final accelerated failure time (AFT) model for chloramphenicol minimum inhibitory concentrations for enterococci isolates (BIC 403.4433, Ggamma distribution).

Factor	Level	Coefficient (SE)	MIC Ratio (SE)	P-value	95% CI
Treatment group	Zinc	0.072 (0.065)	1.07 (0.07)	0.270	0.95, 1.22
	Placebo	Referent	-	-	-
Age of calf (days)	-	-0.015 (0.0096)	0.98 (0.0094)	0.111	0.97, 1.00
Days from last antibiotic treatment	-	-0.0031 (0.011)	1.00 (0.011)	0.775	0.98, 1.02
Intercept	-	2.07 (0.11)	7.93 (0.91)	0	6.33, 9.92

Supplementary Table 12. Final accelerated failure time (AFT) model for daptomycin minimum inhibitory concentrations for enterococci isolates (BIC 522.3062, Weibull distribution).

Factor	Level	Coefficient (SE)	MIC Ratio (SE)	P-value	95% CI
Treatment group	Zinc	0.15 (0.12)	1.16 (0.14)	0.216	0.92, 1.46
	Placebo	Referent	-	-	-
Age of calf (days)		-0.015 (0.0078)	0.98 (0.0077)	0.052	0.97, 1.00
Received spectinomycin treatment	Yes	-0.18 (0.12)	0.84 (0.10)	0.149	0.66, 1.07
	No	Referent	-	-	-
Intercept	-	1.10 (0.14)	2.99 (0.41)	0	2.30, 3.91

Supplementary Table 13. Final accelerated failure time (AFT) model for streptomycin minimum inhibitory concentrations for enterococci isolates (BIC 260.1047, Ggamma distribution).

Factor	Level	Coefficient (SE)	MIC Ratio (SE)	P-value	95% CI
Treatment group	Zinc	0.20 (0.85)	1.22 (1.03)	0.816	0.23, 6.42
	Placebo	Referent	-	-	-
Age of calf (days)	-	-0.20 (0.14)	0.82 (0.11)	0.143	0.62, 1.07
Received spectinomycin treatment	Yes	0.67 (1.09)	1.96 (2.14)	0.537	0.23, 16.68
	No	Referent	-	-	-
Intercept	-	6.61 (1.70)	740.40 (1258.26)	0	26.48, 20702.87

Supplementary Table 14. Final accelerated failure time (AFT) model for tylosin tartrate minimum inhibitory concentrations for enterococci isolates (BIC 390.3383, Weibull distribution).

Factor	Level	Coefficient (SE)	MIC Ratio (SE)	P-value	95% CI
Treatment group	Zinc	1.08 (1.01)	2.94 (3.00)	0.288	0.40, 21.50
	Placebo	Referent	-	-	-
Age of calf (days)	-	-0.0082 (0.12)	0.99 (0.12)	0.946	0.78, 1.26
Days from spectinomycin treatment	-	-0.30 (0.13)	0.74 (0.097)	0.023	0.57, 0.96
Intercept	-	7.93 (1.56)	2779.05	0	130.75, 59066.56

Supplementary Table 15. Final accelerated failure time (AFT) model for quinupristin/dalfopristin minimum inhibitory concentrations for enterococci isolates (BIC 551.3418, Ggamma distribution).

Factor	Level	Coefficient (SE)	MIC Ratio (SE)	P-value	95% CI
Treatment group	Zinc	0.13 (0.13)	1.14 (0.15)	0.340	0.88, 1.47
	Placebo	Referent	-	-	-
Age of calf (days)	-	-0.018 (0.011)	0.98 (0.011)	0.099	0.96, 1.00
Received spectinomycin treatment	Yes	0.20 (0.14)	1.23 (0.17)	0.138	0.94, 1.61
	No	Referent	-	-	-
Intercept	-	0.80 (0.16)	2.24 (0.35)	0	1.64, 3.05

Supplementary Table 16. Final accelerated failure time (AFT) model for linezolid minimum inhibitory concentrations for enterococci isolates (BIC 303.3108, Weibull distribution).

Factor	Level	Coefficient (SE)	MIC Ratio (SE)	P-value	95% CI
Treatment group	Zinc	0.13 (0.076)	1.14 (0.087)	0.079	0.98, 1.33
	Placebo	Referent	-	-	-
Age of calf (days)		0.0048 (0.0070)	1.00 (0.0070)	0.491	0.99, 1.02
Days from last spectinomycin treatment	1-3 days	0.24 (0.098)	1.27 (0.12)	0.016	1.04, 1.53
	4-7 days	0.017 (0.089)	1.02 (0.090)	0.844	0.86, 1.21
	8-23 days	0.044 (0.099)	1.04 (0.10)	0.660	0.86, 1.27
	0 days	Referent	-	-	-
Intercept	-	0.69 (0.11)	2.00 (0.22)	0	1.61, 2.49

Supplementary Table 17. Final accelerated failure time (AFT) model for nitrofurantoin minimum inhibitory concentrations for enterococci isolates (BIC 567.2407, Exponential distribution).

Factor	Level	Coefficient (SE)	MIC Ratio (SE)	P-value	95% CI
Treatment group	Zinc	0.23 (0.25)	1.26 (0.32)	0.354	0.77, 2.06
	Placebo	Referent	-	-	-
Age of calf (days)	-	-0.055 (0.021)	0.95 (0.020)	0.009	0.91, 0.99
Received spectinomycin treatment	Yes	-0.73 (0.28)	0.48 (0.13)	0.008	0.28, 0.83
	No	Referent	-	-	-
Intercept	-	5.42 (0.42)	226.92 (94.47)	0	100.34, 513.15

Supplementary Table 18. Final accelerated failure time (AFT) model for penicillin minimum inhibitory concentrations for enterococci isolates (BIC 644.4164, Exponential distribution).

Factor	Level	Coefficient (SE)	MIC Ratio (SE)	P-value	95% CI
Treatment group	Zinc	0.0061 (0.21)	1.01 (0.21)	0.977	0.67, 1.52
	Placebo	Referent	-	-	-
Age of calf (days)	-	-0.051 (0.013)	0.95 (0.013)	0	0.93, 0.98
Received spectinomycin treatment	Yes	-0.012 (0.21)	0.89 (0.18)	0.569	0.59, 1.33
	No	Referent	-	-	-
Intercept	-	2.57 (0.30)	13.06 (3.92)	0	7.25, 23.52

Supplementary Table 19. Final accelerated failure time (AFT) model for erythromycin minimum inhibitory concentrations for enterococci isolates (BIC 365.5822, Weibull distribution).

Factor	Level	Coefficient (SE)	MIC Ratio (SE)	P-value	95% CI
Treatment group	Zinc	0.90 (1.02)	2.46 (2.52)	0.379	0.33, 18.29
	Placebo	Referent	-	-	-
Age of calf (days)	-	0.0065 (0.12)	1.01 (0.12)	0.955	0.80, 1.26
Days from spectinomycin treatment	-	-0.29 (0.14)	0.75 (0.10)	0.037	0.57, 0.98
Intercept	-	6.40 (1.45)	602.55 (875.78)	0	34.90, 10403.28

Supplementary Table 20. Final accelerated failure time (AFT) model for ciprofloxacin minimum inhibitory concentrations for enterococci isolates (BIC 435.9695, Ggamma distribution).

Factor	Level	Coefficient (SE)	MIC Ratio (SE)	P-value	95% CI
Treatment group	Zinc	0.024 (0.10)	1.02 (0.10)	0.809	0.84, 1.25
	Placebo	Referent	-	-	-
Age of calf (days)	-	-0.019 (0.0082)	0.98 (0.0080)	0.019	0.97, 1.00
Days from spectinomycin treatment	-	0.017 (0.019)	1.02 (0.020)	0.370	0.98, 1.06
Intercept	-	0.74 (0.22)	2.09 (0.47)	0.001	1.35, 3.24

Supplementary Table 21. Final accelerated failure time (AFT) model for vancomycin minimum inhibitory concentrations for enterococci isolates (BIC 451.1961, Ggamma distribution).

Factor	Level	Coefficient (SE)	MIC Ratio (SE)	P-value	95% CI
Treatment group	Zinc	-0.039 (0.099)	0.96 (0.095)	0.694	0.79, 1.17
	Placebo	Referent	-	-	-
Age of calf (days)	-	-0.0049 (0.0074)	1.00 (0.0073)	0.508	0.98. 1.01
Received spectinomycin treatment	Yes	0.089 (0.10)	1.09 (0.11)	0.384	0.89, 1.34
	No	Referent	-	-	-
Intercept	-	-0.56 (0.21)	0.57 (0.12)	0.007	0.38, 0.86

Supplementary Table 22. Final accelerated failure time (AFT) model for gentamicin minimum inhibitory concentrations for enterococci isolates (BIC 524.5724, Exponential distribution).|

Factor	Level	Coefficient (SE)	MIC Ratio (SE)	P-value	95% CI
Treatment group	Zinc	0.37 (0.35)	1.44 (0.51)	0.295	0.73, 2.88
	Placebo	Referent	-	-	-
Age of calf	8-14 days	-1.80 (0.71)	0.17 (0.12)	0.012	0.041, 0.67
	15-21 days	-1.31 (0.82)	0.27 (0.22)	0.111	0.054, 1.35
	23-28 days	-2.63 (0.92)	0.072 (0.066)	0.004	0.012, 0.44
	29-35 days	-0.44 (1.26)	0.64 (0.81)	0.725	0.054, 7.60
	5-7 days	Referent	-	-	-
Days from spectinomycin treatment	-	-0.097 (0.045)	0.91 (0.041)	0.030	0.83, 0.99
Intercept	-	8.75 (0.70)	6331.95 (4440.216)	0	1601.92, 25028.44

Supplementary Table 23. Prediction estimates from final accelerated failure time (AFT) model for azithromycin minimum inhibitory concentrations (MIC, μg/mL) for *E. coli* isolates.

		Predicted MIC (Zinc)				Predicted MIC (Placebo)				Predicted MIC (Difference, Zinc-Placebo)			
		MIC	SE	P-value	95% CI	MIC	SE	P-value	95% CI	MIC	SE	P-value	95% CI
Days from last spectinomycin treatment	0 days	3.33	0.86	0	1.65, 5.02	4.18	1.02	0	2.18, 6.17	-0.84	0.78	0.28	-2.37, 0.69
	3-5 days	8.38	2.11	0	4.24, 12.52	10.50	3.28	0.001	4.07, 16.92	-2.12	2.18	0.33	-6.39, 2.16
	6-8 days	3.42	0.69	0	2.06, 4.78	4.28	0.66	0	2.98, 5.58	-0.86	0.77	0.26	-2.36, 0.64
	9-10 days	9.02	2.01	0	5.09, 12.95	11.3	2.21	0	6.96, 15.63	-2.28	2.07	0.27	-6.33, 1.77

Supplementary Table 24. Prediction estimates from final accelerated failure time (AFT) model for ciprofloxacin minimum inhibitory concentrations (MIC, μg/mL) for *E. coli* isolates.

		Predicted MIC - Zinc				Predicted MIC - Placebo				Predicted MIC (Difference, Zinc-Placebo)			
		MIC	SE	P-value	95% CI	MIC	SE	P-value	95% CI	MIC	SE	P-value	95% CI
Number of spectinomycin doses received	0 doses	0.0024	0.0027	0.37	-0.0029, 0.0077	0.015	0.018	0.42	-0.021, 0.050	-0.012	0.016	0.45	-0.044, 0.020
	1 dose	0.15	0.21	0.48	-0.26, 0.56	0.90	1.12	0.42	-1.29, 3.09	-0.75	0.97	0.44	-2.65, 1.14
	2 dose	0.018	0.013	0.18	-0.0081, 0.044	0.11	0.073	0.14	-0.035, 0.25	-0.091	0.072	0.21	-0.23, 0.05

Supplementary Table 25. Prediction estimates from final accelerated failure time (AFT) model for nalidixic acid minimum inhibitory concentrations (MIC, μg/mL) for *E. coli* isolates.

		Predicted MIC - Zinc				Predicted MIC - Placebo				Predicted MIC – (Difference, Zinc-Placebo)			
		MIC	SE	P-value	95% CI	MIC	SE	P-value	95% CI	MIC	SE	P-value	95% CI
Number of spectinomycin doses received	0 doses	0.69	0.28	0.016	0.13, 1.24	2.47	1.06	0.02	0.39, 4.55	-1.78	0.89	0.044	-3.52, -0.047
	1 dose	5.19	2.63	0.048	0.037, 10.35	18.69	8.55	0.029	1.92, 35.46	-13.50	6.72	0.045	-26.68, -0.32
	2 dose	1.83	0.49	0	0.88, 2.78	6.60	1.58	0	3.50, 9.69	-4.76	1.58	0.002	-7.85, -1.68

Supplementary Table 26. Prediction estimates from final accelerated failure time (AFT) model for ceftriaxone minimum inhibitory concentrations (MIC, μg/mL) for *E. coli* isolates.

		Predicted MIC - Zinc				Predicted MIC - Placebo				Predicted MIC – (Difference, Zinc-Placebo)			
		MIC	SE	P-value	95% CI	MIC	SE	P-value	95% CI	MIC	SE	P-value	95% CI
Received spectinomycin treatment	No	12.26	5.34	0.022	1.79, 22.73	12.97	5.43	0.017	2.32, 23.61	-0.71	4.23	0.87	-9.00, 7.58
	Yes	22.71	6.26	0	10.45, 34.98	24.03	6.54	0	11.21, 36.85	-1.31	7.85	0.87	-16.70, 14.07

Supplementary Table 27. Prediction estimates from final accelerated failure time (AFT) model for tigecycline minimum inhibitory concentrations (MIC) for enterococci isolates.

		Predicted MIC - Zinc				Predicted MIC - Placebo				Predicted MIC – (Difference, Zinc-Placebo)			
		MIC	SE	P-value	95% CI	MIC	SE	P-value	95% CI	MIC	SE	P-value	95% CI
Days from last spectinomycin treatment	0 days	0.31	0.03	0.00	0.25, 0.38	0.30	0.03	0.00	0.24, 0.36	0.02	0.02	0.47	-0.03, 0.06
	1 days	0.32	0.03	0.00	0.25, 0.39	0.31	0.03	0.00	0.24, 0.37	0.02	0.02	0.47	-0.03, 0.06
	5 days	0.36	0.04	0.00	0.28, 0.44	0.34	0.04	0.00	0.26, 0.42	0.02	0.03	0.47	-0.03, 0.07
	10 days	0.41	0.06	0.00	0.30, 0.52	0.39	0.05	0.00	0.28, 0.49	0.02	0.03	0.47	-0.04, 0.08

Supplementary Table 28. Prediction estimates from final accelerated failure time (AFT) model for chloramphenicol minimum inhibitory concentrations (MIC) for enterococci isolates.

		Predicted MIC - Zinc				Predicted MIC - Placebo				Predicted MIC – (Difference, Zinc-Placebo)			
		MIC	SE	P-value	95% CI	MIC	SE	P-value	95% CI	MIC	SE	P-value	95% CI
Days from last spectinomycin treatment	0 days	8.52	0.95	0	6.65, 10.38	7.93	0.91	0	6.14, 9.71	0.59	0.54	0.271	-0.46, 1.65
	1 days	8.49	0.99	0	6.56, 10.43	7.90	0.94	0	6.06, 9.75	0.59	0.54	0.271	-0.46, 1.64
	5 days	8.39	1.19	0	6.06, 10.71	7.80	1.12	0	5.61, 10.00	0.58	0.53	0.273	-0.46, 1.63
	10 days	8.26	1.51	0	5.31, 11.21	7.69	1.41	0	4.91, 10.46	0.57	0.53	0.277	-0.46, 1.61

Supplementary Table 29. Prediction estimates from final accelerated failure time (AFT) model for daptomycin minimum inhibitory concentrations (MIC) for enterococci isolates.

		Predicted MIC - Zinc				Predicted MIC - Placebo				Predicted MIC – (Difference, Zinc-Placebo)			
		MIC	SE	P-value	95% CI	MIC	SE	P-value	95% CI	MIC	SE	P-value	95% CI
Received spectinomycin treatment	No	3.47	0.63	0.00	2.25, 4.70	2.99	0.41	0.00	2.20, 3.79	0.48	0.42	0.26	-.34, 1.30
	Yes	2.90	0.39	0.00	2.13, 3.67	2.50	0.36	0.00	1.79, 3.22	0.40	0.32	0.22	-.23, 1.03

Supplementary Table 30. Prediction estimates from final accelerated failure time (AFT) model for streptomycin minimum inhibitory concentrations (MIC) for enterococci isolates.

		Predicted MIC - Zinc				Predicted MIC - Placebo				Predicted MIC – (Difference, Zinc-Placebo)			
		MIC	SE	P-value	95% CI	MIC	SE	P-value	95% CI	MIC	SE	P-value	95% CI
Received spectinomycin treatment	No	901.75	1718.45	0.60	-2466.34, 4269.85	740.40	1258.26	0.56	-1725.75, 3206.55	161.35	814.67	0.84	-1435.37, 1758.07
	Yes	1769.14	3045.05	0.56	-4199.05, 7737.33	1452.58	2355.66	0.54	-3164.43, 6069.59	316.56	1516.10	0.84	-2654.93, 3288.05

Supplementary Table 31. Prediction estimates from final accelerated failure time (AFT) model for tylosin tartrate minimum inhibitory concentrations (MIC) for enterococci isolates.

		Predicted MIC – Zinc				Predicted MIC - Placebo				Predicted MIC – (Difference, Zinc-Placebo)			
		MIC	SE	P-value	95% CI	MIC	SE	P-value	95% CI	MIC	SE	P-value	95% CI
Days from last spectinomycin treatment	0 days	8176.12	10455.33	0.43	-12315.95, 28668.19	2779.05	4333.91	0.52	-5715.27, 11273.36	5394.07	7686	0.48	-9667.21, 20461.36
	1 days	6081.64	8088.78	0.45	-9772.08, 21935.36	2067.14	3382.43	0.54	-4562.31, 8696.58	4014.5	5823.56	0.49	-7399.47, 15428.47
	5 days	1861.72	3027.34	0.54	-4071.76, 7795.19	632.79	1265.48	0.62	-1847.49, 3133.08	1228.92	2037.77	0.55	-2765.04, 5222.89
	10 days	423.92	899.63	0.64	-1339.33, 2187.16	144.09	365.00	0.69	-571.31, 859.48	279.83	581.72	0.63	-860.32, 1419.97

Supplementary Table 32. Prediction estimates from final accelerated failure time (AFT) model for quinupristin/dalfopristin minimum inhibitory concentrations (MIC) for enterococci isolates.

		Predicted MIC – Zinc				Predicted MIC - Placebo				Predicted MIC – (Difference, Zinc-Placebo)			
		MIC	SE	P-value	95% CI	MIC	SE	P-value	95% CI	MIC	SE	P-value	95% CI
Received spectinomycin treatment	No	2.54	0.52	0.00	1.52, 3.56	2.24	0.35	0.00	1.54, 2.93	0.30	0.34	0.37	-0.36, 0.97
	Yes	3.11	0.62	0.00	1.90, 4.33	2.74	0.51	0.00	1.75, 3.74	0.37	0.40	0.36	-0.42, 1.16

Supplementary Table 33. Prediction estimates from final accelerated failure time (AFT) model for linezolid minimum inhibitory concentrations (MIC) for enterococci isolates.

		Predicted MIC – Zinc				Predicted MIC - Placebo				Predicted MIC – (Difference, Zinc-Placebo)			
		MIC	SE	P-value	95% CI	MIC	SE	P-value	95% CI	MIC	SE	P-value	95% CI
Days from last spectinomycin treatment	0 days	2.29	0.24	0.00	1.82, 2.75	2.00	0.22	0.00	1.57, 2.44	0.29	0.16	0.08	-0.03, 0.61
	1-3 days	2.90	0.30	0.00	2.31, 3.48	2.53	0.32	0.00	1.90, 3.17	0.36	0.20	0.07	-0.02, 0.75
	4-7 days	2.33	0.30	0.00	1.75, 2.91	2.04	0.28	0.00	1.48, 2.59	0.29	0.16	0.08	-0.03, 0.62
	8-23 days	2.39	0.33	0.00	1.75, 3.04	2.09	0.36	0.00	1.38, 2.80	0.30	0.16	0.05	-0.0042, 0.60

Supplementary Table 34. Prediction estimates from final accelerated failure time (AFT) model for nitrofurantoin minimum inhibitory concentrations (MIC) for enterococci isolates.

		Predicted MIC – Zinc				Predicted MIC – Placebo				Predicted MIC – (Difference, Zinc-Placebo)			
		MIC	SE	P-value	95% CI	MIC	SE	P-value	95% CI	MIC	SE	P-value	95% CI
Received spectinomycin treatment	No	286.38	136.52	0.04	18.80, 553.95	226.92	94.47	0.02	41.76, 412.08	59.46	74.99	0.43	-87.52, 206.44
	Yes	138.08	52.36	0.01	35.45, 240.70	109.41	36.06	0.002	38.74, 180.08	28.67	34.35	0.40	-38.66, 96.00

Supplementary Table 35. Prediction estimates from final accelerated failure time (AFT) model for penicillin minimum inhibitory concentrations (MIC) for enterococci isolates.

		Predicted MIC - Zinc				Predicted MIC - Placebo				Predicted MIC – (Difference, Zinc-Placebo)			
		MIC	SE	P-value	95% CI	MIC	SE	P-value	95% CI	MIC	SE	P-value	95% CI
Received spectinomycin treatment	No	13.14	4.22	0.002	4.87, 21.41	13.06	3.92	0.001	5.38, 20.74	0.08	2.74	0.98	-5.29, 5.45
	Yes	11.69	2.98	0.00	5.85, 17.53	11.62	3.06	0.00	5.62, 17.61	0.07	2.44	0.98	-4.70, 4.85

Supplementary Table 36. Prediction estimates from final accelerated failure time (AFT) model for erythromycin minimum inhibitory concentrations (MIC) for enterococci isolates.

		Predicted MIC - Zinc				Predicted MIC - Placebo				Predicted MIC – (Difference, Zinc-Placebo)			
		MIC	SE	P-value	95% CI	MIC	SE	P-value	95% CI	MIC	SE	P-value	95% CI
Days from last spectinomycin treatment	0 days	1481.63	1765.71	0.40	-1979.09, 4942.36	602.55	875.78	0.49	-1113.94, 2319.05	879.08	1290.93	0.50	-1651.09, 3409.24
	1 days	1111.13	1352.30	0.41	-1539.32, 3761.58	451.88	683.07	0.51	-886.92, 1790.67	659.25	964.63	0.49	-1231.38, 2549.89
	5 days	351.45	509.79	0.49	-647.73, 1350.63	142.93	261.76	0.59	-370.12, 655.98	208.52	326.92	0.52	-432.23, 849.28
	10 days	83.37	161.12	0.61	-232.43, 399.16	33.90	79.82	0.67	-122.55, 190.35	49.46	95.23	0.60	-137.19, 236.12

Supplementary Table 37. Prediction estimates from final accelerated failure time (AFT) model for ciprofloxacin minimum inhibitory concentrations (MIC) for enterococci isolates.

		Predicted MIC - Zinc				Predicted MIC - Placebo				Predicted MIC – (Difference, Zinc-Placebo)			
		MIC	SE	P-value	95% CI	MIC	SE	P-value	95% CI	MIC	SE	P-value	95% CI
Days from last spectinomycin treatment	0 days	2.15	0.51	0.00	1.15, 3.15	2.09	0.47	0.00	1.18, 3.01	0.05	0.21	0.81	-0.37, 0.47
	1 days	2.18	0.54	0.00	1.12, 3.24	2.13	0.50	0.00	1.15, 3.11	0.05	0.22	0.81	-0.37, 0.48
	5 days	2.34	0.69	0.001	0.98, 3.70	2.28	0.66	0.00	1.00, 3.57	0.06	0.23	0.81	-0.40, 0.51
	10 days	2.55	0.95	0.007	0.70, 4.40	2.49	0.91	0.006	0.70, 4.28	0.06	0.25	0.81	-0.44, 0.56

Supplementary Table 38. Prediction estimates from final accelerated failure time (AFT) model for vancomycin minimum inhibitory concentrations (MIC) for enterococci isolates.

		Predicted MIC - Zinc				Predicted MIC - Placebo				Predicted MIC – (Difference, Zinc-Placebo)			
		MIC	SE	P-value	95% CI	MIC	SE	P-value	95% CI	MIC	SE	P-value	95% CI
Received spectinomycin treatment	No	0.55	0.12	0.00	0.32, 0.78	0.57	0.12	0.00	0.34, 0.81	-0.02	0.06	0.69	-0.13, 0.09
	Yes	0.60	0.12	0.00	0.37, 0.83	0.63	0.12	0.00	0.40, 0.85	-0.02	0.06	0.69	-0.14, 0.09

Supplementary Table 39. Prediction estimates from final accelerated failure time (AFT) model for gentamicin minimum inhibitory concentrations (MIC) for enterococci isolates.

		Predicted MIC - Zinc				Predicted MIC - Placebo				Predicted MIC – (Difference, Zinc-Placebo)			
		MIC	SE	P-value	95% CI	MIC	SE	P-value	95% CI	MIC	SE	P-value	95% CI
Days from last spectinomycin treatment	0 days	9147.40	6884.36	0.18	-4345.70, 22640.50	6331.95	4440.22	0.15	-2370.71, 15034.62	2815.45	3602.90	0.44	-4246.11, 9877.00
	1 days	8300.11	6209.46	0.18	-3870.20, 20470.42	5745.45	4037.86	0.16	-2168.61, 13659.50	2554.66	3242.34	0.43	-3800.21, 8909.53
	5 days	5626.37	4258.12	0.19	-2719.40, 13972.14	3894.65	2869.00	0.18	-1728.48, 9517.78	1731.72	2151.63	0.42	-2485.39, 5948.83
	10 days	3460.66	2851.98	0.23	-2129.12, 9050.45	2395.52	1995.06	0.23	-1514.74, 6305.77	1065.15	1326.07	0.42	-1533.896, 3664.187
